# Processing and analysis of portable EEG data for cognitive load assessment in neurotypical university students

**DOI:** 10.3389/fnhum.2026.1737723

**Published:** 2026-03-19

**Authors:** María Consuelo Sáiz-Manzanares, Raúl Ortega-Renuncio, Raúl Marticorena-Sánchez

**Affiliations:** 1 Departamento de Ciencias de la Salud, Universidad de Burgos, Burgos, Spain; 2 Escuela Politécnica Superior, Universidad de Burgos, Burgos, Spain; 3 Departamento de Ingeniería Informática, Universidad de Burgos, Burgos, Spain

**Keywords:** cognitive load, EEG data processing, electroencephalogram (EEG), higher education, metacognition

## Abstract

The use of electroencephalogram (EEG) to gain insight into cognitive and metacognitive processing during task execution is being pioneered in natural learning contexts; an opportunity not without its challenges. Accordingly, a pilot study was conducted to explore the feasibility of this approach. The aims of this study were: (1) to demonstrate how raw data extracted from an EEG device may be processed; (2) to determine whether there were differences in pre-task cognitive load between senior university students (Group 1), novice university teachers (Group 2) and experienced university teachers (Group 3); (3) To determine whether the peak power (μV^2^) per brain band (Delta, Theta, Alpha, Beta and Gamma) recorded during task performance was different depending on the type of participant; (4) To determine whether there were un-labelled groupings (clusters), and whether they corresponded to the type of participant. The raw data were processed using the MNE-Python toolkit. No significant differences were found in the perception of cognitive load or in peak power with respect to participant type. However, different frequencies of maximum activation of brain channels in the Delta wave were found by participant type. The largest overlaps were found between Group 1 and Group 2. Future studies will address the influence of other variables such as age, gender, type of studies and cranial tomography. In addition, 3D analyses with integration of brain surfaces and sensors will be applied.

## 1 Introduction

One of the primary aims of education is to develop learners’ thinking skills—particularly creative thinking skills—that enable them to address future personal and professional challenges. Information processing during learning involves multiple cognitive and metacognitive processes, such as attention, working memory, and the application of cognitive and metacognitive strategies (Sáiz-Manzanares et al., 2023). These processes are influenced by both internal and external factors, implying that access to information may be facilitated or inhibited by emotional components (Schunk and DiBenedetto, 2020).

Technological advances now allow these processes, together with associated emotional responses, to be recorded through various devices, including eye-tracking systems, galvanic skin response (GSR) sensors, and electroencephalography (EEG) (Sáiz-Manzanares et al., 2024). In educational settings, EEG provides insights into cognitive and metacognitive processes during task execution. Nevertheless, employing EEG data to characterize learning processes remains a challenge that offers significant opportunities but also presents methodological and interpretive difficulties.

The main opportunities lie in the objective, empirical measurement of information processing during learning (Sáez-García et al., 2024). For instance, EEG can quantify the cognitive load imposed by a given task (Sáiz-Manzanares et al., 2024). Such information may be used to determine the most efficient processing strategies across tasks or to optimize task presentation according to individual learners’ processing profiles. These advances represent a significant contribution to neuroscience, cognitive science, and instructional psychology, as the results enable more personalized learning approaches (Beauchemin et al., 2024; Dan and Reiner, 2017; Sarailoo et al., 2022). From a neurotechnological perspective, EEG can also support neurofeedback applications (Kopańska et al., 2022; Kopańska et al., 2023; Kopańska et al., 2024), fostering improvements in cognitive and metacognitive strategies and ultimately enhancing learning outcomes (Jamil et al., 2021).

Although early EEG systems were considered invasive, modern devices are less intrusive and designed for use in naturalistic contexts (Rivas et al., 2025). Depending on the model, EEG signals are recorded through electrodes that may be dry or semidry. Dry electrodes are simpler to use and often embedded in lightweight hardware, such as headbands or adhesive strips. Despite these improvements, data analysis and interpretation remain major challenges. Raw EEG signals require pre-processing and subsequent processing steps (Bigdely-Shamlo et al., 2015; Pion-Tonachini et al., 2019).

EEG data report the intensity of neural activity, i.e., the electrical activity generated by groups of neurons synchronized under each electrode, measured in microvolts (μV). These data can also be transformed from the time domain into the frequency domain (cycles per second of brain activity), providing information about the activation of distinct brain rhythms (Delta, Theta, Alpha, Beta, and Gamma). Interpreting these metrics yields information on signal amplitude (μV), signal power or energy (μV^2^), and spectral power, which describes the distribution of power across frequency bands. This indicates the energy associated with each frequency range during task-related processing. A summary of the metrics, their units of measurement, and their interpretation is provided in Table 1.

**Table 1:** Metrics, units of measurement and meaning.

Concept	Unit	Meaning
Amplitude	μV	Signal voltage magnitude (signal size)
Power	μV^2^	Signal-related energy
Exchange rate	μV/s	Fast voltage changeover

This represents an important milestone in the field of cognitive and instructional psychology. Information regarding the activation of different brain areas makes it easier to understand the type of processing being applied, depending on both the task and the learner profile (Fink et al., 2009). Supplementary Table S1 presents the relationship between brain area signal recordings and their implications for information processing analysis (consulted in Supplementary Table S1). These signals are interpreted using the international 10–20 system, a standardized method for positioning and labeling electrodes in electroencephalography (EEG), which defines their placement on the scalp for consistent measurement and analysis.

As mentioned above, from the signal recorded in microvolts (μV), its frequency can be obtained in hertz (Hz), which provides information about the wavelength during execution of a task. It can also provide information about a learner’s level of activation during a task (see Table 2 for the relationship between brainwaves, their interpretation, cognitive state, and their role in processing visual stimuli).

**Table 2:** Relationship between brainwaves, their meaning, cognitive state and interpretation of visual stimuli.

Brain waves	Interpretation	Cognitive status	Role in processing visual stimuli
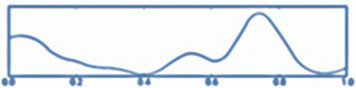 Delta waves (δ)	Delta waves (*δ*) are the result of the representation of brain activity versus sleep time. They have a periodicity in the frequency range of 0.5–4 Hz.	It is associated with deep dreamless sleep (NREM sleep). Regeneration and consolidation processes.	Activity of states of low consciousness. May increase in states of visual fatigue or attentional disengagement.
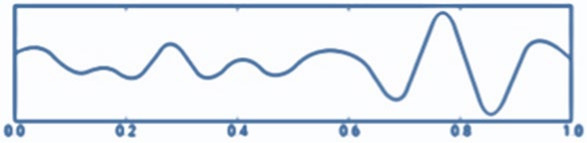 Theta (θ)	Theta waves (θ) refer to activity between 4 and 8 Hz. Waves in the theta range are generally considered abnormal in the adult EEG during wakefulness; however, the appearance of theta waves is one of the hallmarks of the onset of normal sleepiness.	Working memory, encoding and retrieval of memories. States of drowsiness, reverie or light meditation. Emotional processing.	Appears when visual processing with memory load is required. It has been observed in the identification of visual objects or stimulus changes. It is also related to creative processes or visual insight.
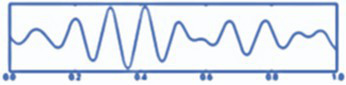 Alpha waves (α)	Alpha (*α*) waves are electromagnetic oscillations in the frequency range 8 Hz-13 Hz that arise from synchronous, coherent electrical activity. They originate mainly in the occipital lobe during periods of relaxation, with the eyes closed, but still in the waking state.	State of relaxation with eyes closed. Cortical inhibition (filtering out irrelevant information). Suppression mechanism to protect internal tasks from the external environment.	Decreases (Alpha suppression) when the eyes are opened or a visual stimulus is presented. Alpha reduction in occipital regions indicates active visual attention. Higher Alpha suppression may indicate efficient attentional focus.
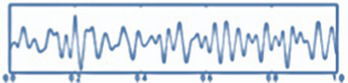 Beta (β) waves	Beta (β) waves are electromagnetic oscillations in the second highest frequency range 13–30 Hz.	It is associated with: Alertness, sustained attention, logical thinking. Motor activity and movement control.	Increases when the subject is concentrating on a visual stimulus. Participates in visual decision-making, especially when there is ambiguity or judgement. More visible in complex visual tasks or tasks involving immediate feedback.
Gamma waves (γ) 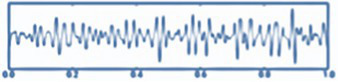	Gamma (γ) waves are a neuronal oscillation pattern that occurs in humans, ranging in frequency from 30 Hz-100 Hz, although the most common presentation is 40 Hz.	Sensory integration: help to unify visual information from different parts of the visual field (e.g., shape, color, movement). Visual attention: these are heightened when the brain focuses on a relevant visual stimulus (attentional focus). Conscious processing: Elevated gamma activity is related to conscious perception of images, beyond simple detection. Pattern recognition: Involved in the recognition of objects, faces and letters, facilitating perceptual coherence. Integration, awareness, attention.	Conscious perception, image integration.

EEG interfaces can also facilitate objective monitoring of individuals’ cognitive states, such as cognitive load (Spüler et al., 2016). Current EEG interfaces focus mainly on the spectral aspects of EEG, i.e., the type of neural oscillations, or “brain waves,” that can be observed in EEG signals (Niedermeyer and da Silva, 2004). The challenge is to develop techniques that allow real-time processing and interpretation of the data to gain insight into the learner’s cognitive state (Spüler et al., 2016). For example, according to cognitive load theory (Sweller et al., 1998), increasing task difficulty should increase cognitive load and thus brain activity in frontal (domain-general structures) and parietal (domain-specific structures) areas. This means that an increase in cognitive load should result in an increase in EEG power in the Theta band and a decrease in the Alpha band (Pesonen et al., 2007). The Theta and Alpha bands have been found to be the most predictive of cognitive load (Spüler et al., 2016). For example, when looking at facial expressions, the facial fusiform area is involved in processing these stimuli (Mourão-Miranda et al., 2005). In particular, time-averaged and time-synchronized measurement distances after visual stimuli give rise to different components of the power curve (Metzen et al., 2025). Recent studies using EEG devices have found that the measurement and assessment of the learning process with respect to students’ cognitive states is closely related to oscillatory activity in the theta and alpha frequency bands (Li et al., 2017; Nguyen et al., 2020). Theta wave activity has been consistently identified as a robust predictor of changes in cognitive demands, particularly reflecting frontal lobe engagement during executive control, attention allocation, and working memory processes (Grunwald et al., 2014; Roux and Uhlhaas, 2014). Complementarily, alpha-band activity has been associated with attentional modulation and information processing efficiency, with alpha desynchronization typically reflecting increased task engagement, whereas alpha synchronization has been linked to internally oriented processing and creative cognition (Fink et al., 2009). Beyond task-related cognitive demands, recent EEG research indicates that theta and alpha oscillations are also sensitive to the emotional content of visual stimuli, including the perception of emotionally salient images. Emotional processing has been shown to elicit increased theta activity, reflecting integrative and evaluative processes, alongside alpha desynchronization, particularly in posterior and frontal regions, indicating enhanced attentional allocation to emotionally relevant information. These findings suggest that cognitive load and emotional processing are dynamically intertwined and jointly modulate theta–alpha activity during complex learning tasks. Overall, the use of EEG devices provides valuable insights into learners’ diverse cognitive strategies and mental states when engaging with different tasks. However, task performance and neural responses are also influenced by individual characteristics such as age, gender, educational background, and professional experience (Sweller, 2024). Moreover, cognitive, behavioral, affective, and environmental factors, as well as methodological aspects including task design and EEG device characteristics, can significantly impact the interpretation of neural markers of learning and cognitive load (Jamil et al., 2021; Sweller, 2024).

In addition, Machine Learning techniques must be applied to analyze EEG recordings, such as supervised classification techniques to differentiate between different levels of task difficulty. Supervised prediction techniques can also be used (Rivas et al., 2025) (allowing identification of the most significant cognitive demands during task resolution, for example, the oscillations in the Theta and Alpha bands recorded in the parietal electrodes reflect greater difficulty experienced by the learner doing the task) (Spüler et al., 2016). Unsupervised Machine Learning techniques such as clustering may also be applied to EEG signal analysis. This approach is particularly useful, as it allows discovery of hidden patterns and latent structures in brain data (Xiong et al., 2020). For example, it has been used to study EEG data on emotion recognition (Bengalur et al., 2023; Zhang et al., 2023). Similarly, Othman et al. (2021) applied the *k*-Means algorithm to analyze features of EEG signals to detect situational interest in learning environments, demonstrating the applicability of clustering in educational contexts.

### 1.1 Challenges in EEG signal processing

As indicated initially, this technology does present significant challenges. Measuring students’ attention during the learning process is still a complex task, but objective resources such as EEG offer more accurate and, more importantly, non-subjective measurements than those provided by off-line methods (self-reports, questionnaires), as the subject’s perception always has a high degree of subjectivity (Veenman et al., 2014). However, signal processing is complex and therefore a major challenge when it comes to using these devices. This is because the data are usually obtained in a raw form, i.e., unprocessed, and to draw accurate conclusions the data have to be first pre-processed (cleaned and sorted) and then processed (treated computationally). The EEG signal is collected in μV, i.e., voltage over time, then a sampling frequency is applied (which in this study was 256 Hz), then the signal must be filtered and cleaned in order to remove non-neural artefacts (in this study it was at 40 Hz), then the artefacts of eye movement, blinks, etc. are removed [e.g., Principal Component Analysis (PCA) and Independent Component Analysis (ICA)]. Subsequently, a spectral analysis (transformation from the time domain to the frequency domain) is performed by applying the Fourier Transform (FFT) or a Wavelet Transform. In this study we applied FFT (see formula).


$$F(w)=\int_{-\infty }^{\infty }f(t)e^{-\mathrm{i\omega t}}\mathrm{dt}$$

F(w) is equal to the Fourier transform.

f(t) is the signal in time.

*i* is the imaginary unit.

ω is the angular frequency.

*dt* infinitesimal increment of the variable 𝑡 (derivative of *t*).

Reducing dimensionality and scaling of EEG features is also essential for efficient analysis. This is typically achieved using Principal Component Analysis (PCA), which identifies orthogonal directions in the data that maximize variance, thereby simplifying the dataset, and Independent Component Analysis (ICA), which isolates statistically independent components rather than merely correlated ones. Applying these methods helps produce cleaner and more representative EEG data.

Accuracy is a critical goal throughout this process (Rehman et al., 2025). Several multi-platform tools support EEG processing, including EEGLAB and MNE. EEGLAB, developed at the University of California, San Diego, is an interactive MATLAB-based environment widely used in cognitive neuroscience and neuroengineering (Delorme and Makeig, 2004). It supports multiple data formats and enables pre-processing tasks such as bandpass filtering, artefact correction, bad channel interpolation, epoch segmentation, event rejection, and time-frequency analysis, with 2D and 3D visualizations of power spectra. MNE is an open-source Python library for MEG and EEG data processing, analysis, and visualization (Gramfort et al., 2014). It allows import of various data formats, supports raw, epoch, and averaged data, and provides functions for filtering, artefact removal, epoch manipulation, averaging, time-frequency and power/phase analysis, as well as 3D visualization integrating brain surfaces and sensors. Despite their popularity and functionality, both EEGLAB and MNE require programming skills for effective use.

### 1.2 Interpretation of EEG metrics

As discussed above, EEG devices record signals that generate various metrics. Raw data are measured in microvolts (μV), reflecting the instantaneous amplitude of the electrical signal. For instance, the P300 waveform may have an amplitude of 8 μV, providing information about signal strength at a specific moment. This measure is useful for analyzing peaks, latencies, and waveforms (Martinez-Cagigal et al., 2017). Another important metric is μV^2^, which quantifies the power or energy of the signal over time. Power metrics are typically derived using spectral analysis (e.g., Fourier or Wavelet transforms) and are applied in comparisons across conditions (such as pre- and post-stimulus) or as indicators of brain and muscle activation. These metrics are obtained after appropriate filtering and processing of the data. Recent studies, however, have questioned the rigid assignment of specific brain functions to electrode localization within the international 10–20 EEG system. Scrivener and Reader (2022) reported substantial inter-subject variability in electrode positioning and anatomical correspondence, which may challenge strictly localized interpretations of brain activity. Similarly, Ignatiadis et al. (2022) showed that both individual anatomy and electrode placement significantly influence source localization accuracy in the primary auditory cortex, highlighting the need to account for individual differences in EEG analyses. Advanced methodologies for three-dimensional and automated electrode localization, as described by Taberna et al. (2019) and Bhutada et al. (2020), further underscore the importance of precision when attributing functions to specific brain regions. Additionally, Opitz et al. (2021) demonstrated that individual anatomical variability affects the efficacy of stimulation techniques such as transcranial Direct Current Stimulation (tDCS), while Chella et al. (2016) highlighted that reference choice significantly impacts EEG connectivity patterns. Collectively, these findings suggest that brain functions cannot be universally assigned to specific electrode locations, indicating the need for more dynamic and individualized approaches in contemporary neuroscience research.

### 1.3 Objectives and research questions

In light of the above, the overall objective of this study is to examine how EEG data can be applied, processed, and interpreted within a higher education context in order to provide insights into students’ performance during the execution of an avatar visualization task representing different emotional states.

Specifically, the study aims to: (i) analyze perceived cognitive workload among three participant groups—final-year undergraduate students, junior university lecturers, and senior university lecturers—while considering the effects of age group and gender; (ii) examine whether the power or energy of the electrical signal (μV^2^) across different brainwave frequency bands (Delta, Theta, Alpha, Beta, and Gamma) recorded during task performance varies according to participant type, age group, and gender; and (iii) explore whether unlabeled clusters derived from EEG and cognitive workload measures correspond to participant type.

Based on these objectives, four research questions were formulated to illustrate how EEG, together with appropriate raw data processing and analytical methods, can inform researchers and educational practitioners about cognitive and affective processes in higher education learning contexts.

*RQ1*: To determine whether perceived cognitive workload (NASA-TLX subscales) differs between participant types (final-year students, adjunct lecturers, and full professors), controlling for age group and gender.

*RQ2:* To determine whether EEG power across frequency bands (Delta, Theta, Alpha, Beta, Gamma) differs between participant types, controlling for age group and gender.

*RQ3:* To determine whether unsupervised clusters derived from perceived cognitive workload and mean EEG power across frequency bands correspond to participant type.

*RQ4:* To determine whether differences exist in the maximum EEG power recorded at specific scalp sensors (AF7, FP1, FP2, AF8, F3, F4, P3, P4, PO7, O1, O2, PO8) according to participant type, based on qualitative frequency analysis.

## 2 Methods

### 2.1 Participants

We worked with a convenience sample of 22 participants in 3 groups: Group 1: students in the final year of their undergraduate degree (final-year students) (*n* = 11), average age range: 20–30 years, 9.1% men and 90.9% women; Group 2: university teachers with less than 4 years of experience (junior lecturers) (*n* = 6), average age range: 20–30 years, 100% women; and Group 3: university teachers with more than 15 years of professional experience (senior lecturers) (*n* = 5), average age range: 42–52 years, 40% men and 60% women. All participation was informed, voluntary and without compensation.

### 2.2 Instruments

#### 2.2.1 Images representing emotions

The study used five images, developed *ad hoc*, of different freely accessible emotions (Sáiz-Manzanares, 2024). These images depicted an avatar representing different emotions. The aim was to check whether there were different cognitive reactions to the different avatars and to check whether these differed depending on the type of user (students in the final year of their undergraduate degree, junior lecturers, senior lecturers). These results will be used in the future design of educational agents to support learning.

#### 2.2.2 NASA-TLX (Task Load Index)

The NASA-TLX is a subjective assessment instrument that measures the workload perceived by a person when performing a task (Hart and Staveland, 1988). NASA-TLX assesses 6 dimensions: Mental Load (mental and perceptual effort required); Physical Load (physical effort required); Time Load (time pressure or urgency to complete the task); Performance (subjective evaluation of success in performing the task); Effort (total amount of physical and mental work required) and Frustration Level (feelings of insecurity, stress or irritation). Each dimension is scored on a scale from 0 to 100. The scale can be applied in a weighted or unweighted version. In the weighted version, each participant compares each pair of dimensions (15 in total) and chooses which of the two is considered the most relevant workload for that task, the value obtained being the weighting value. Next, the participant rates each of the 6 dimensions on a scale of 0–100. The value obtained is then multiplied by the weighting value.


$$\text{NASA}-\mathrm{TLX}=\frac{\sum (\text{Scor}e_{i}x\;\text{Weightin}g_{i})}{15}$$

In the unweighted application only, the simple average is applied and the paired comparisons step is omitted.


$$\text{NASA}-\mathrm{TLX}\;(\text{simplified})=\frac{\sum \text{Score}}{6}$$

The NASA Task Load Index (NASA-TLX) is a widely used subjective instrument for assessing perceived workload, encompassing dimensions such as mental demand, effort, frustration, and temporal demand (Hart and Staveland, 1988). Although originally developed for complex control and operational tasks, its applicability has been extended to perceptual, visual, and emotional tasks, in which participants must process stimuli, maintain sustained attention, and make rapid decisions (Recarte et al., 2008; Brouwer et al., 2012).

In the present study, participants viewed images representing different emotions, which entails perceptual processing, emotional categorization, and potential affective regulation, all of which consume limited cognitive resources (Pessoa, 2008, 2009; Schupp et al., 2006). The perceived cognitive workload is complemented by objective neurophysiological measures obtained via EEG, which have been shown to correlate with subjective NASA-TLX scores in tasks involving attention and visual processing (Berka et al., 2007; Galy et al., 2012).

Therefore, the NASA-TLX is an appropriate tool for capturing the subjective experience of cognitive workload in emotional processing tasks, allowing for a comprehensive analysis combining subjective and objective indicators.

#### 2.2.3 Headset

**Figure 1:**
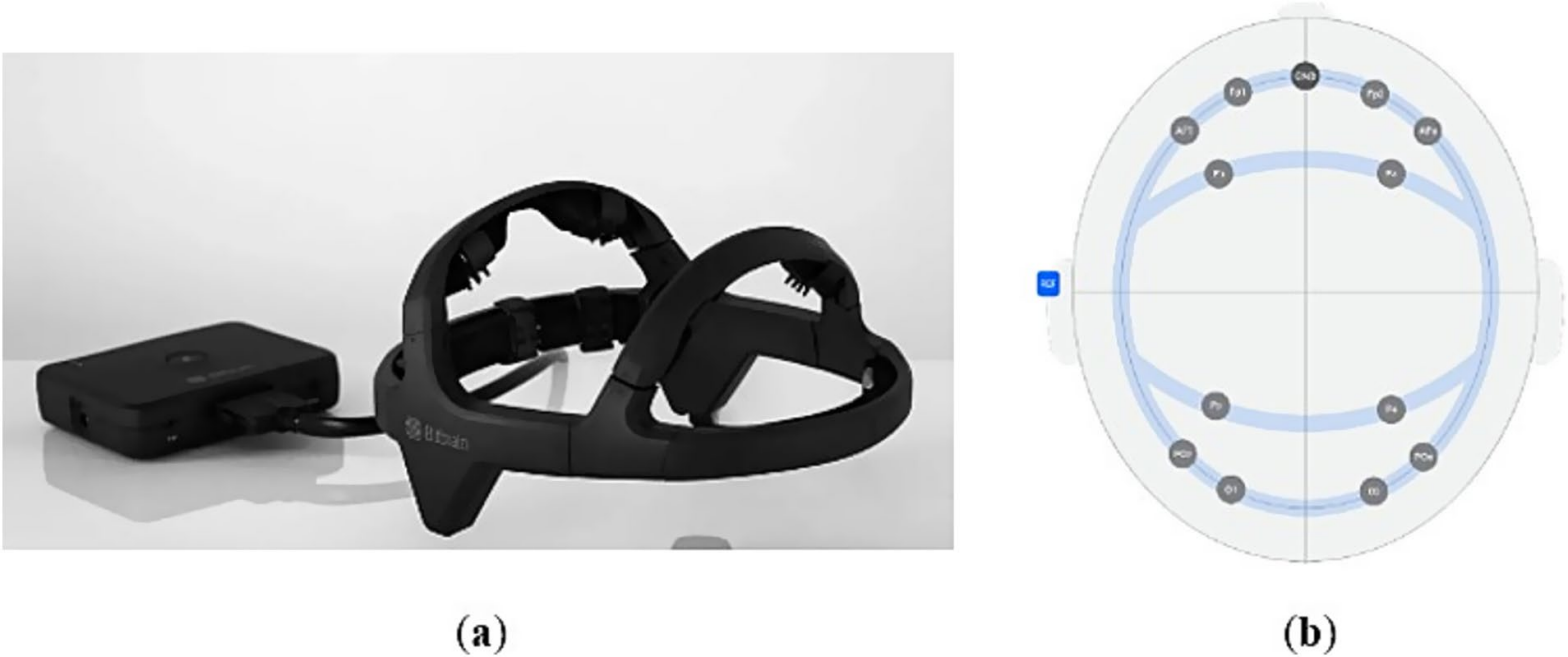
**(a)** Bitbrain headband. **(b)** Arrangement of the 12 electrodes.

Dry-sensor EEG optimized to monitor emotional and cognitive states in real-world applications (Bitbrain, 2025, Zaragoza, Spain). It is lightweight (190 g) and flexible, allowing it to adapt to the morphology of each head (85% of the global population). It has 12 dry sensors and a design that reduces impedances and ensures stable contacts, along with active shielding to maximize SNR. It also has a small, lightweight (125 g) mobile amplifier that records with 24-bit resolution at 256 Hz. It has an internal IMU (9 DOF), and online relative impedance control. It can record to microSD or over Bluetooth at a range of over 10 m, and provides up to 8 h of continuous recording. It comes with a software package and SDK for programmers, and is compatible with Matlab, Python, etc. The device is shown in Figure 1.

The Bitbrain EEG headset provides objective neurophysiological measures of cognitive workload by recording electrical brain activity associated with attention, working memory, and information processing. Cognitive workload has been shown to modulate specific EEG markers, such as theta and alpha band power, event-related potentials (ERPs), and frontal–parietal activation patterns (Berka et al., 2007; Gevins and Smith, 2003; Klimesch, 2009).

In the present study, participants performed an emotional image visualization task, which, despite not being analytically complex, engages perceptual processing, attentional control, emotional categorization, and potential affective regulation. These processes consume limited cognitive resources, which are detectable in EEG recordings. The Bitbrain headset has been validated in research settings for capturing these neural signatures of workload, showing reliable correlation with both task demands and subjective measures of mental effort (Brouwer et al., 2012; Galy et al., 2012).

### 2.3 Procedure

The procedure followed for the study is described below:

*Step 1*. Approval for the study was obtained from the Bioethics Commission of the University of Burgos (No. IO 5/2024).

*Step 2*. Written informed consent was obtained from all participants.

*Step 3*. First, the NASA-TLX scale was applied in its weighted form.

**Figure 2:**
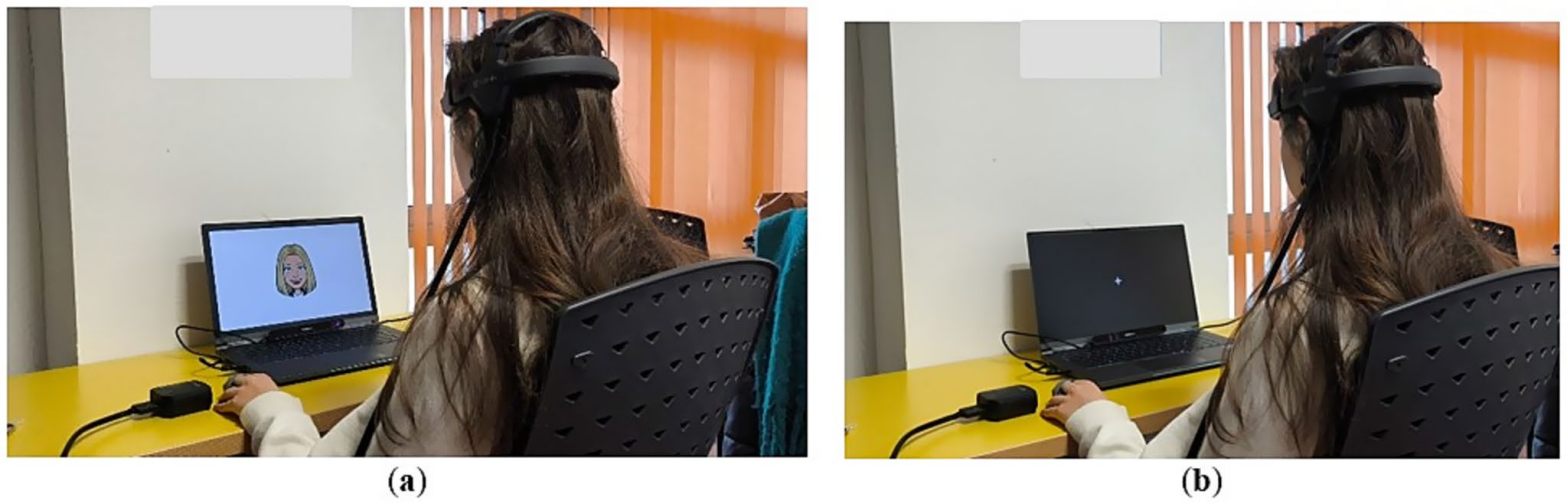
**(a)** Image display. **(b)** Rest stimulus between image displays.

*Step 4*. The experimental phase was carried out using the Bitbrain headband EEG device. This phase consisted of presenting the subjects with five images representing different emotions. Each image was displayed for 6 s, and between each image, a rest stimulus (lasting 5 s) and a white cross on a black background (lasting 3 s) were shown. An example is shown in Figure 2.

*Step 5.* Pre-processing to remove artefacts and noise. This was done using the Python programming language, and a notebook was created in Jupyter where the following steps were executed:

*Step 5.1.* The following libraries and modules were imported: pandas (Python library specializing in data analysis); NumPy (Python library specializing in numerical computation for data analysis); matplotlib (Python data visualization library); SciPy (mathematics, science and engineering library built on top of NumPy); MNE (Minimum Norm Estimation), an open source Python module for processing, analyzing and visualizing functional neuroimaging data (EEG, MEG, ECoG, and fNIRS).

*Step 5.2.* Definition of electrodes and frequency bands [“Delta”: (1, 4), “Theta”: (4, 8), “Alpha”: (8, 12), “Beta”: (12, 30), “Gamma”: (>30)] to define the wavelength range per band, Webster’s classification (Webster, 2020) was applied using Bitbrain’s recommended band-pass filter from 1 to 40 Hz.

*Step 5.3*. Load Data:

- Data loaded and prepared; EEG event times and times loaded from CSV files.
$$X(t)=[x_{1}(t),x_{12}(t)\ldots ,x_{N}(t)]$$

*Step 5.4*. Initial configuration:

- Converted microvolts to volts and transposed.
$$V_{i}(t)=\frac{\mu V_{i}(t)}{10^{6}}$$
- Created channel information.
- Configured assembly.

*Step 6.* Pre-processing:

- Pre-processing of EEG data by 1–40 Hz band-pass filtering and artefact removal by ICA.
$$X_{\text{filt}}(t)=X_{t}*h_{t}$$

$$\begin{array}{l} \text{where}\;h_{t}\;\;\text{is the impulse response of the band}-\text{pass filter},\text{and}\;\mathrm{its}\;\\ \text{frequency response is}: \end{array}$$

$$H(f)=\{\begin{array}{c} 1\;\;\;\;\text{if}\;\;1\leq f\leq 40\\ 0\;\;\;\text{otherwise} \end{array}\}$$

Independent Component Analysis (ICA)

X=AS

where

X=observedEEGsignals

A=mixing matrix

S=independent components

After artifact removal

$$X_{\text{clean}}=AS_{\text{corr}}$$

where

$$\begin{array}{c} S_{\text{corr}}=\text{excludes artifact}-\text{related componets}\;(e.g.,\;\mathrm{eye}\;\text{blinks},\\ \text{muscle activity}). \end{array}$$

*Step 7.* Time acquisition for each image:

- Dictionary created to store consecutive image values and their corresponding EEG segments.
$$T_{\mathrm{ima}}(i)=[t_{\text{start}}^{i},t_{\mathrm{end}}^{i}\;]$$

$$\mathrm{EE}G_{\mathrm{ima}}(i)=X_{t}\;\text{for}\;t\in T_{\mathrm{ima}}(i)$$

*Step 8.* Frequency processing:

- Power in the frequency bands calculated.
$$X_{i}(f)=F\{x_{i}(t)\}$$
- Power spectral density calculated.
$$P_{i}(f)={|X_{i}(f)|}^{2}$$

Band Power

$$P_{\text{band},i}=\int_{f_{1}}^{f_{2}}P_{i}(f)\mathrm{df}$$

*Step 9.* Data extraction.

- Power for each band calculated.
$${\bar{P}}_{\text{band}}=\frac{1}{N_{c}}\;\sum_{i=1}^{N_{c}}P_{\text{band},i}$$

$$\text{ where}\;N_{c}\;\text{ is the number of channels}$$

**Figure 3:**
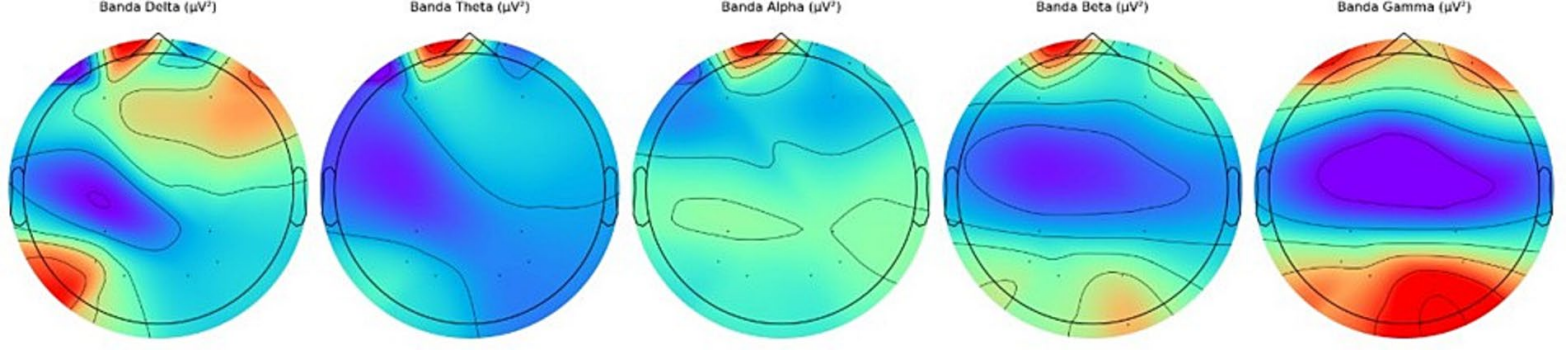
Cerebral tomography representation.

*Step 10.* Visualization (see Figure 3):

- Visualizations produced.
- EEG raw chart.
- Topographical maps.
- Descriptive graphs.
- Power per band.

*Step 11.* Statistical data

- Band power calculations.

*Step 12.* Save results:

- Results saved to a CSV file.

In sum, EEG activity was recorded using a 12-channel dry-sensor Bitbrain headset (Bitbrain, Zaragoza, Spain) optimized for monitoring cognitive and emotional states. The device records at 256 Hz with 24-bit resolution, employs active shielding to maximize signal-to-noise ratio (SNR), and uses online relative impedance control. Participants wore the headset while viewing emotional images, each presented for 6 s with interspersed rest periods.

Raw EEG data were preprocessed using MNE-Python v1.5.1. A zero-phase FIR band-pass filter (1–100 Hz, Hann window, 53 dB stopband) was first applied to remove slow drifts and high-frequency noise, followed by average referencing across channels. Independent Component Analysis (ICA) using the fastica algorithm was applied to remove ocular and muscular artefacts, with an average of 3.2 ± 0.9 components removed per participant and no channels rejected. Residual noisy segments were excluded, resulting in 7.5 ± 3.2% of data rejection. After preprocessing, the cleaned EEG signals exhibited an average standard deviation of 4.3 μV across channels, indicating low-noise and stable recordings. A second FIR band-pass filter (1–45 Hz) was applied post-ICA to finalize the signal for analysis.

EEG epochs were defined relative to each stimulus, spanning from −0.2 to +1.0 s, with the first 50 ms excluded to remove transient artefacts associated with stimulus onset. Stimulus timestamps were converted from the recorded absolute timestamps to sample indices based on the EEG sampling frequency. Across participants, this procedure resulted in 9 usable epochs per subject. Baseline activity was controlled using a pre-stimulus interval from −2 to 0 s.

Also, frequency analysis was performed for each epoch using Welch’s method with 512-sample windows, 50% overlap, and a Hann window. Power spectral density (PSD) was averaged for canonical frequency bands: Delta (1–4 Hz), Theta (4–8 Hz), Alpha (8–12 Hz), Beta (12–30 Hz), and Gamma (>30 Hz), and normalized relative to the pre-stimulus baseline using percentage change to ensure that spectral measures reflected stimulus-induced activity. These frequency-domain measures provided objective indicators of cognitive workload, complementing subjective assessments from the NASA-TLX questionnaire (Berka et al., 2007; Brouwer et al., 2012; Galy et al., 2012).

**Figure 4:**
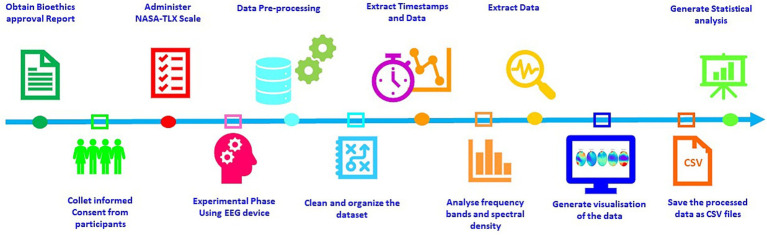
Raw data processing with minimum norm estimation (MNE).

All steps were required in order for the analysis of the datasets (Md Nor et al., 2022). The procedure is summarized in Figure 4.

### 2.4 Design and data analysis

The study employed a cross-sectional, descriptive–correlational design (Campbell and Stanley, 2005). Research questions RQ1 and RQ2 were addressed using a quantitative approach, RQ3 was addressed using a computational-exploratory clustering approach, and RQ4 was addressed using a comparative frequency-based approach.

Prior to interpreting the results of the MANOVA, the multivariate assumptions of homogeneity and normality were considered. Rather than testing normality for each dependent variable individually, the value of Pillai’s Trace was used to assess the overall multivariate effect. Pillai’s Trace is robust to moderate violations of multivariate normality and provides a global indication of whether the combination of dependent variables is significantly influenced by the independent factors. Only when Pillai’s Trace indicated a significant multivariate effect were follow-up univariate analyses conducted.

To address RQ1, perceived cognitive workload was analyzed using a three-factor between-subjects multivariate analysis of variance (MANOVA), with the NASA-TLX subscales entered as dependent variables and participant type, age group, and gender specified as fixed factors. Given the relatively small sample size, Pillai’s Trace was used to assess multivariate effects due to its robustness to violations of multivariate normality. When significant multivariate effects were observed, follow-up univariate analyses were conducted for each NASA-TLX subscale, with Bonferroni-adjusted post-hoc comparisons applied where appropriate. Effect sizes (partial eta squared, η*_p_*^2^) were reported only for the univariate analyses.

For RQ2, EEG power (μV^2^) in the Delta, Theta, Alpha, Beta, and Gamma bands was analyzed using a multivariate general linear model (MANOVA), with participant type specified as the main fixed factor, and age group and gender included as additional fixed factors. A multivariate approach was adopted due to the continuous and correlated nature of the EEG frequency bands. Multivariate effects were assessed using Pillai’s Trace, and when significant, follow-up univariate analyses with Bonferroni-adjusted post-hoc comparisons were conducted. Effect sizes (partial eta squared, η*_p_*^2^) were reported only for the univariate analyses. Effect sizes were interpreted according to Cohen (1988), whereby partial eta squared values (η*_p_*^2^) of 0.01 ≤ η*_p_*^2^ < 0.06 were considered small, 0.06 ≤ η*_p_*^2^ < 0.14 moderate, and η*_p_*^2^ ≥ 0.14 large.

To address RQ3, an unsupervised *k*-means clustering analysis was performed using perceived cognitive workload and mean EEG power across Delta, Theta, Alpha, Beta, and Gamma bands as variables. The optimal number of clusters (k) was determined using the elbow method. Subsequently, the correspondence between the resulting clusters and participant type was evaluated using contingency tables and chi-square tests, complemented by measures of association.

RQ4 was analyzed using frequency-based methods, including contingency tables and the contingency coefficient, to examine differences in maximum EEG power recorded at scalp sensors (AF7, FP1, FP2, AF8, F3, F4, P3, P4, PO7, O1, O2, PO8) according to participant type. Age group and gender were not included, as doing so would extend the scope of the study beyond its primary objectives and substantially increase the number of comparisons.

A summary of the relationship between the research questions (RQs) and data processing can be found in Table 3.

**Table 3:** Summary of the tests performed in the studies and data analysis software used for each analysis.

Quantitative study	Contrast tests	Data analysis software
RQ1	Three-factor between-subjects MANOVA with fixed effects (participant type, age group, gender); partial eta squared (η*_p_*^2^) reported for univariate analyses	Statistical package SPSS v.31 (IBM Corp, 2016)
RQ2	MANOVA with participant type as fixed factor, controlling for age group and gender; partial eta squared (η*_p_*^2^) reported for univariate analyses	Statistical package SPSS v.31 (IBM Corp, 2016)
RQ3	*k*-means cluster analysis; optimal number of clusters determined using the elbow method; cluster-participant group correspondence assessed with contingency tables	Python library (McKinney, 2010) Statistical package SPSS v.31 (IBM Corp, 2016)
RQ4	Frequency-based analysis using contingency tables and the contingency coefficient	Data Mining Software Orange v. 3.38.1 (Demšar et al., 2013)

## 3 Results

### 3.1 Testing research questions

- To test research questions RQ1 and RQ2, a multivariate analysis of variance (MANOVA) with fixed effects was performed. Participant type was included as the main independent variable (Group 1: final-year students; Group 2: junior lecturers; Group 3: senior lecturers), and age group and gender were included as additional factors to control for their potential influence.

For RQ1, to determine whether perceived cognitive workload (NASA-TLX subscales) differs between participant types (final-year students, adjunct lecturers, and full professors), controlling for age group and gender. Non-significant Pillai’s Trace values were observed for all three factors (participant type, age group, and gender), indicating that the groups did not differ multivariately on the NASA-TLX subscales. No statistically significant differences were found for any of the dependent variables. However, descriptive effect sizes (partial eta squared, η*_p_*^2^) suggested moderate effects for participant type on Effort (η*_p_*^2^ = 0.21), Performance (η*_p_*^2^ = 0.11), Frustration (η*_p_*^2^ = 0.21), and Weighted Workload (η*_p_*^2^ = 0.21) (see Table 4). For age group, descriptive η*_p_*^2^ values suggested moderate effects on Physical Demand (η*_p_*^2^ = 0.12), Effort (η*_p_*^2^ = 0.15), Performance (η*_p_*^2^ = 0.20), Frustration (η*_p_*^2^ = 0.15), and Weighted Workload (η*_p_*^2^ = 0.14). In contrast, descriptive effect sizes for gender were small across all variables (see Table 4).

**Table 4:** MANOVA results for NASA-TLX subscales in Phase 2, with participant type as the fixed factor, controlling for age group and gender.

Nasa-TLX dimension	Group 1 *n* = 11	Group 2 *n* = 5	Group 3 *n* = 6	*F*	*p*	*η_p_* ^2^
Factor: participant type						
Mental demand	47.12(23.3)	42(16.4)	40(23.6)	0.23	0.80	0.03
Physical demand	29.5(17.1)	32(17.9)	25(13.8)	0.05	0.95	0.01
Temporal demand	42.8(25.3)	40(18.7)	38.3(11.7)	0.18	0.84	0.03
Effort	65.5(21.6)	44(25.1)	70(24.5)	1.77	0.21	0.21
Performance	69.4(23.0)	78(16.5)	80(8.9)	0.82	0.46	0.11
Frustration	65.5(21.6)	44(25.1)	70(24.5)	1.77	0.21	0.21
Weighted workload	21.3(5.1)	18.7(6.2)	21.6(5.5)	1.31	0.30	0.17
Factor: age						
Mental demand	47.12(23.3)	42(16.4)	40(23.6)	0.07	0.94	0.01
Physical demand	29.5(17.1)	32(17.9)	25(13.8)	0.86	0.45	0.12
Temporal demand	42.8(25.3)	40(18.7)	38.3(11.7)	0.19	0.83	0.03
Effort	65.5(21.6)	44(25.1)	70(24.5)	1.10	0.36	0.15
Performance	69.4(23.0)	78(16.5)	80(8.9)	1.58	0.24	0.20
Frustration	65.5(21.6)	44(25.1)	70(24.5)	1.10	0.36	0.15
Weighted workload	21.3(5.1)	18.7(6.2)	21.6(5.5)	1.07	0.37	0.14
Factor: gender						
Mental demand	47.12(23.3)	42(16.4)	40(23.6)	0.03	0.87	0.00
Physical demand	29.5(17.1)	32(17.9)	25(13.8)	0.04	0.84	0.00
Temporal demand	42.8(25.3)	40(18.7)	38.3(11.7)	0.60	0.45	0.04
Effort	65.5(21.6)	44(25.1)	70(24.5)	0.09	0.76	0.01
Performance	69.4(23.0)	78(16.5)	80(8.9)	0.00	0.98	0.00
Frustration	65.5(21.6)	44(25.1)	70(24.5)	0.09	0.76	0.01
Weighted workload	21.3(5.1)	18.7(6.2)	21.6(5.5)	0.00	0.95	0.00

*p ≤ 0.05; Group 1: final-year students; Group 2: junior lecturers; Group 3: senior lecturers.

These results do not support inferential conclusions but point to descriptive trends that may be relevant for future studies with larger samples or different experimental designs.

With regard to RQ2, no significant differences in power (μV2) were found between the three groups of participants. Prior to further analyses, Pillai’s Trace values were examined. No significant multivariate differences were observed for the factors participant type or age group, whereas a significant multivariate effect was found for gender, indicating that EEG power (μV^2^) in the Delta, Theta, Alpha, and Beta bands differed between genders. However, the associated effect sizes ranged from small to moderate, suggesting a limited practical impact. No significant differences were found for the factor participant type, and the effect sizes were small. For the factor age group, no significant differences were observed, although moderate effects were found in the Delta (η*_p_*^2^ = 0.15) and Beta (η*_p_*^2^ = 0.15) bands. Regarding the factor gender, significant differences were found in the Delta, Theta, Alpha, and Beta bands, with effect sizes ranging from moderate to large (see Table 5).

**Table 5:** MANOVA results for brainwave frequency, with participant type as the fixed factor, controlling for age group and gender (both categorical factors).

Mean power (μV^2^)	Group 1 *n* = 11	Group 2 *n* = 5	Group 3 *n* = 6	*F*	*p*	*η^2^*
Factor: participant type						
Delta	614.68(570.34)	364.58(256.51)	2332.64(3995.97)	0.25	0.78	0.04
Theta	113.44(109.48)	70.69(40.89)	170.99(166.55)	0.60	0.56	0.08
Alpha	23.61(23,51)	13.05(4.97)	27.60(22.41)	0.47	0.64	0.07
Beta	11.83(7.1)	11.88(6.70)	20.03(12.18)	0.50	0.62	0.07
Gamma low	0.21(0.14)	0.28(0.34)	0.33(0.23)	0.03	0.97	0.00
Factor: age						
Delta	612.92(637.64)	364.59(265.51)	2332.65(3695.97)	0.87	0.44	0.12
Theta	113.44(109.48)	70.69(40.89)	170.99(166.55)	0.63	0.55	0.09
Alpha	23.61(23,51)	13.05(4.97)	27.60(22.41)	0.39	0.69	0.06
Beta	11.83(7.1)	11.88(6.70)	20.03(12.18)	1.59	0.24	0.20
Gamma low	113.44(109.48)	70.69(40.89)	170.99(166.55)	0.63	0.55	0.09
Factor: gender						
Delta	612.92(637.64)	364.59(265.51)	2332.65(3695.97)	4.80	0.05*	0.27
Theta	113.44(109.48)	70.69(40.89)	170.99(166.55)	9.51	0.01*	0.42
Alpha	23.61(23,51)	13.05(4.97)	27.60(22.41)	6.13	0.03*	0.32
Beta	11.83(7.1)	11.88(6.70)	20.03(12.18)	8.39	0.01*	0.39
Gamma low	113.44(109.48)	70.69(40.89)	170.99(166.55)	0.74	0.41	0.05

*p ≤ 0.05. Group 1: final-year students; Group 2: junior lecturers; Group 3: senior lecturers.

These results may be relevant for future studies with larger samples or alternative experimental designs.

**Figure 5:**
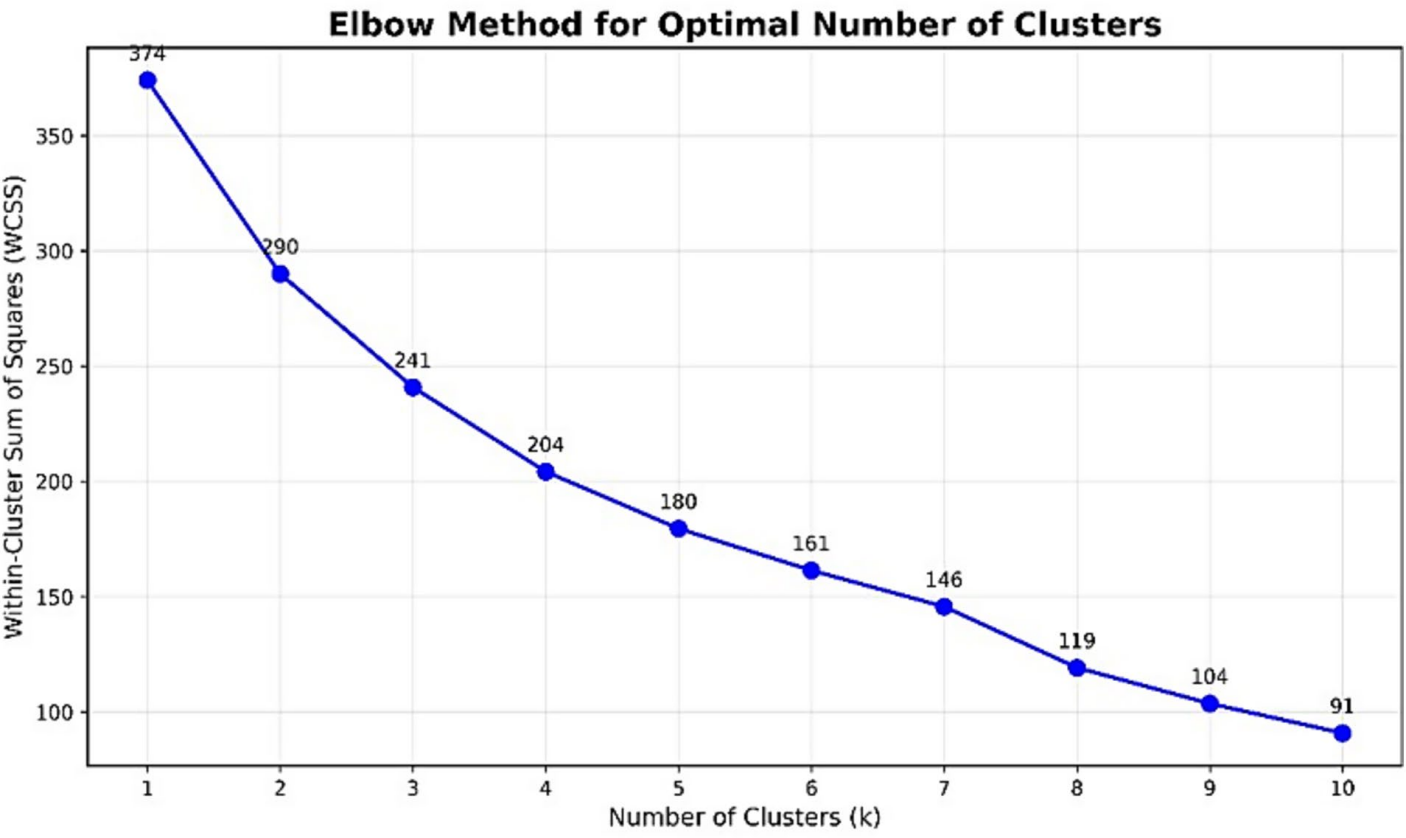
Elbow method for optimal number of clusters.

- With regard to RQ3, three clusters were established, based on the results of the elbow test (see Figure 5), between which significant differences were detected in the Delta, Theta, Alpha, and Beta band (see Table 6).
- Looking at the relationship between the clusters participants were in and the groups they belonged to, a Contingency Index of C = 0.43 was obtained, which was not significant (*p* = 0.28). The group with the highest correspondence was Group 2, junior lecturers (100%), followed by Group 1 (final-year students) where the percentage of correspondence was 81.85% in cluster 1. With regard to Group 3 (senior lecturers), 60% were in cluster 1, 20% in cluster 2 and 20% in cluster 3 (see Table 7).

**Table 6:** One-factor fixed-effects ANOVA (participant type) on the cognitive load data in NASA-TLX phase 2.

Band	Cluster 1 *n* = 18	Cluster 2 *n* = 1	Cluster 3 *n* = 3	*F*	*p*
Delta	380.55	8857.75	1634.82	510.61	<0.001*
Theta	70.02	420.49	252.04	19.21	<0.001*
Alpha	15.93	48.86	46.77	5.99	0.010*
Beta	11.15	37.41	21.18	11.03	<0.001*
Gamma	0.22	0.71	0.37	3.38	0.055

*p ≤ 0.05.

**Table 7:** Relationship between clusters and their relationship with the factor participant type.

Participants	Cluster 1 n (%)	Cluster 2 n (%)	Cluster 3 n (%)	Total
Final-year students	9(81.85)	0(0)	2(18.15)	11
Junior lecturers	6(100)	0(0)	0(0)	6
Senior lecturers	3(60)	1(20)	1(20)	5
Total	18(81.1)	1(4.55)	3(13.63)	22

To explore patterns in spectral power across participants and channels, *k*-means clustering was applied to the normalized band-power features. Prior to clustering, spectral power values were z-score normalized across channels and frequency bands to remove scale differences and ensure comparability. The number of clusters, *k* = 3, was selected based on the elbow method, balancing within-cluster variance and interpretability. Given the relatively small sample size (*n* = 22), this unsupervised clustering approach was applied with an explicitly exploratory aim: to descriptively examine potential correspondences between frequency bands and electrode locations, rather than to identify stable, generalizable, or inferential group differences.

In Cluster 1, which comprised the majority of participants, the strongest activity was observed in the theta band at the Fp1 channel, with a descriptive tendency for higher activity in participant Groups 1 and 3. Cluster 2, which included a single participant, was characterized by activity in the delta and theta bands at the Fp1 channel. Cluster 3, comprising three participants, showed prominent activity across multiple frequency bands, including delta, theta, alpha, and beta, mainly at the F4 channel. Additional activity was observed at the O1 channel, potentially reflecting visual processing. A descriptive correspondence with participant Group 2 was observed in the gamma band at the PO8 channel.

These observations represent trends in spectral activity across channels rather than precise functional or anatomical localization. The results are intended as exploratory and descriptive, providing insight into potential patterns in the data while avoiding overinterpretation of cognitive processes or brain functions at specific electrode sites (consulted in Supplementary Table S2).

Relating to RQ4, in the Delta frequency band, the highest spectral power was observed in the Fp1 channel for Group 1 (45.45%) and Group 2 (66.6%), while in Group 3 it was observed equally in the F4 and Fp1 channels (40% each). In the Theta band, the highest activity was in Fp1 for Group 1 (54.54%), in F4 for Group 2 (66.66%), and in F4 and Fp1 for Group 3 (40% each). For the Alpha band, Group 1 showed peak activity at Fp1 (36.4%), Group 2 at O2 (33.3%), and Group 3 across multiple channels (O1, F4, AF8, Fp2, PO7; 20% each). In the Beta band, peak activity was observed at PO8 for Group 1 (36.36%), at O2 for Group 2 (33.33%), and across multiple channels in Group 3 (O1, F4, AF8, Fp2, PO7; 20% each). In the Gamma band, Group 1 showed the highest activity at Fp1 and O1 (27.3% each), Group 2 at Fp1 (50%), and Group 3 across multiple channels (O1, PO8, AF8, Fp1, PO7; 20% each) (consulted in Supplementary Table S3) summarizes these observations across channels and participant groups.

**Figure 6:**
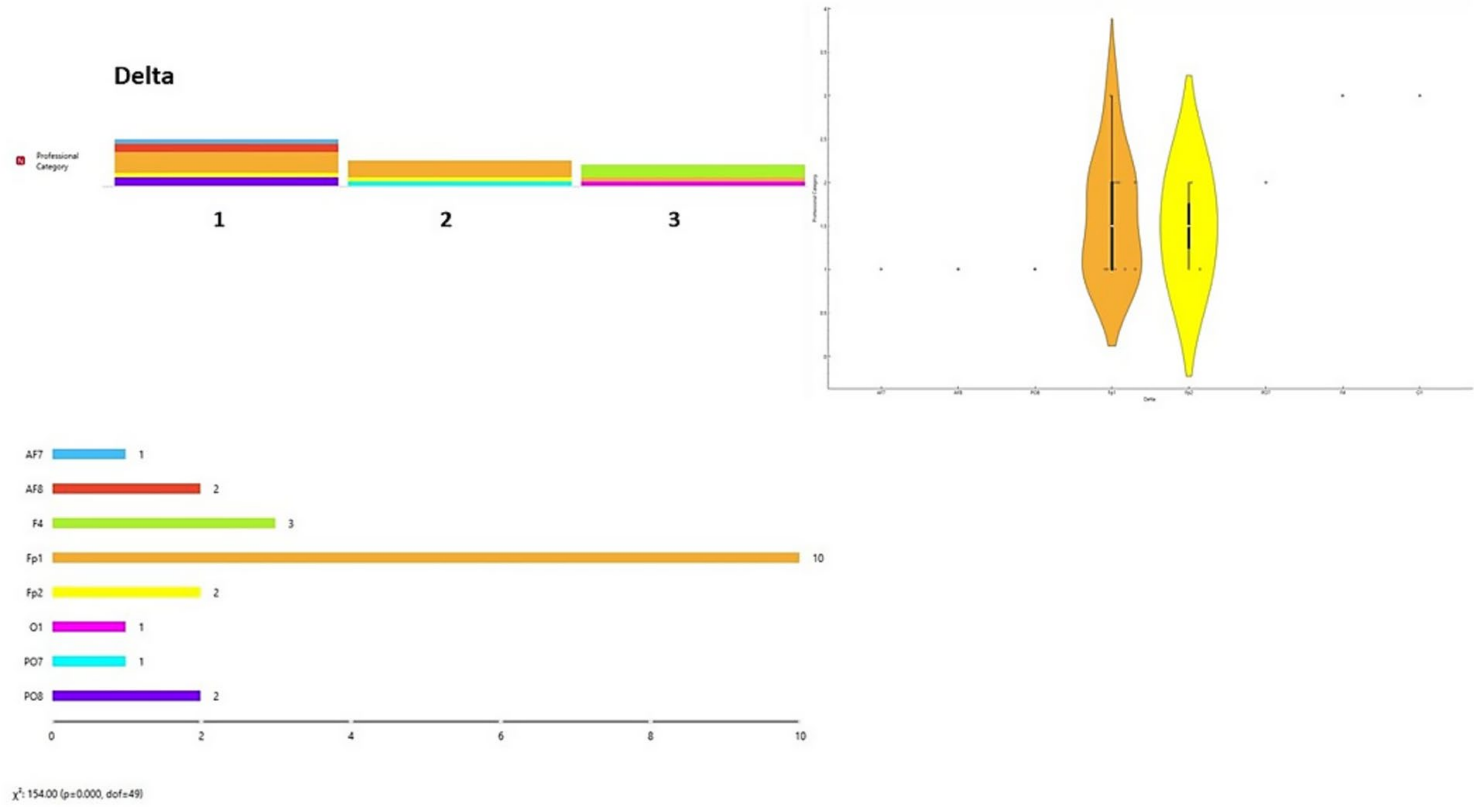
Delta wave analysis of the distribution of frequency of use in brain channels and Chi-square test (χ^2^). 1 = Group 1: final-year students; Group 2: junior lecturers; Group 3: senior lecturers.

**Figure 7:**
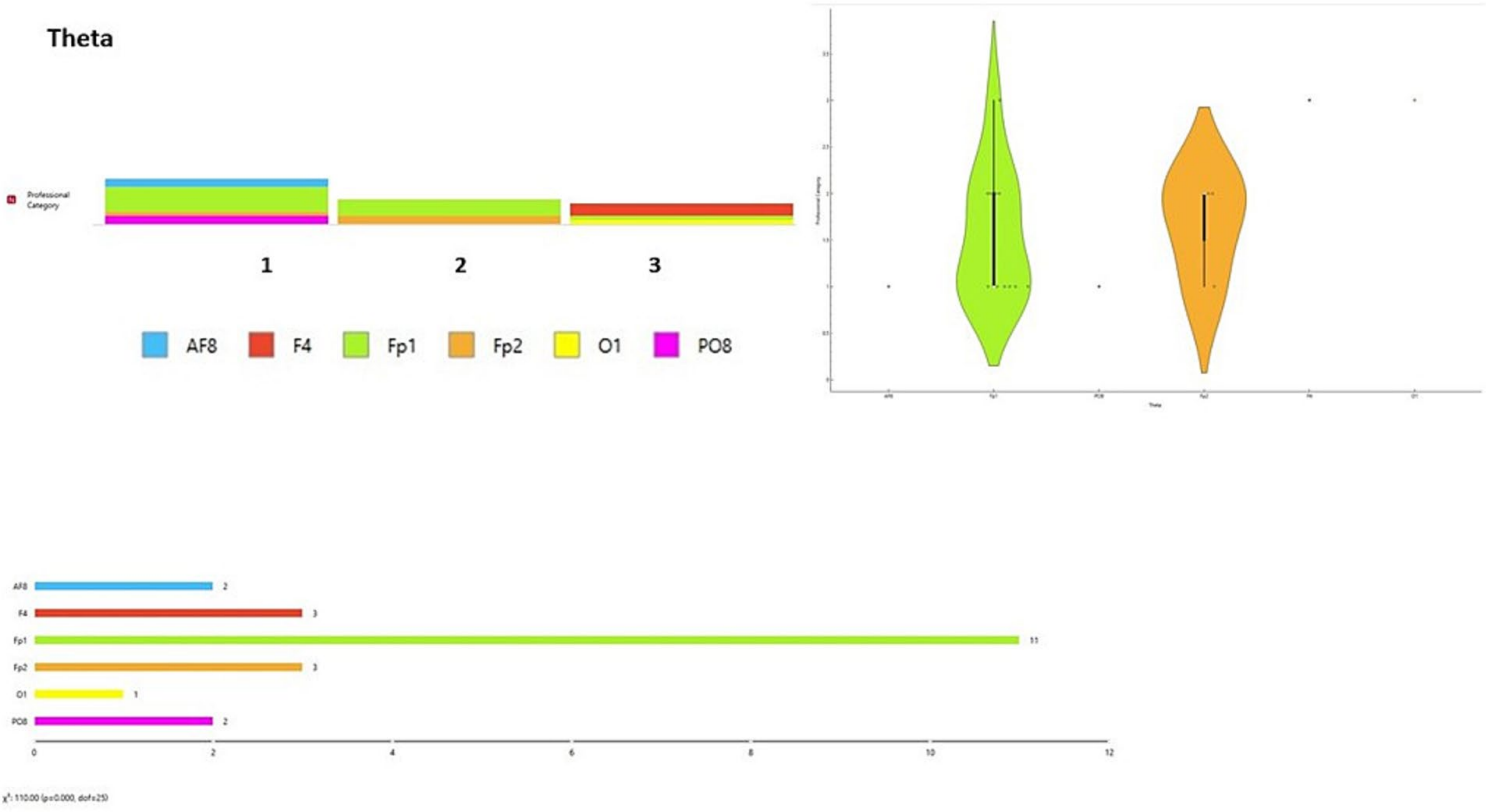
Theta wave analysis of the distribution of frequency of use in brain channels and Chi-square test (χ^2^). 1 = Group 1: final-year students; Group 2: junior lecturers; Group 3: senior lecturers.

**Figure 8:**
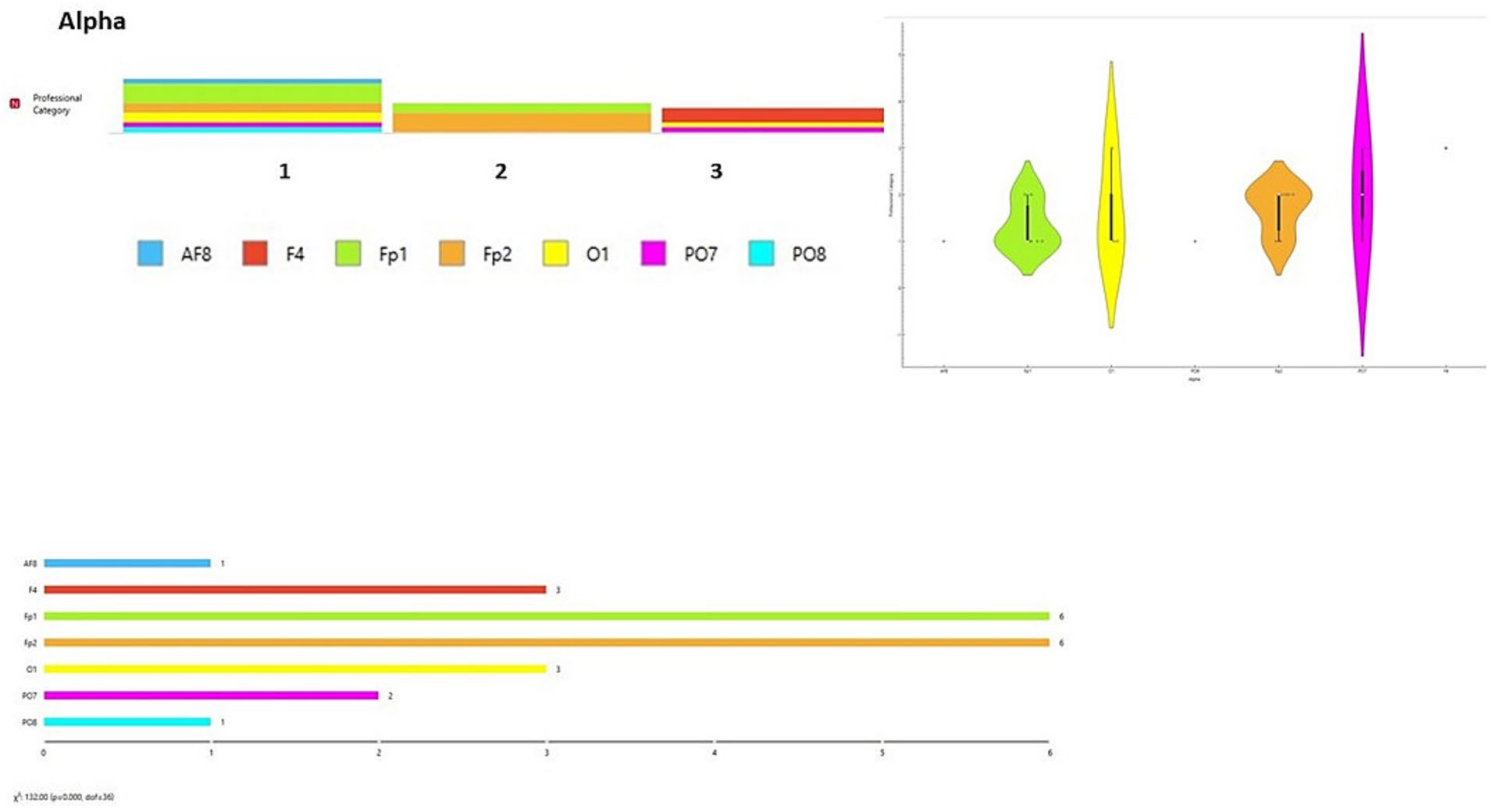
Alpha wave analysis of the distribution of frequency of use in brain channels and Chi-square test (χ^2^). 1 = Group 1: final-year students; Group 2: junior lecturers; Group 3: senior lecturers.

**Figure 9:**
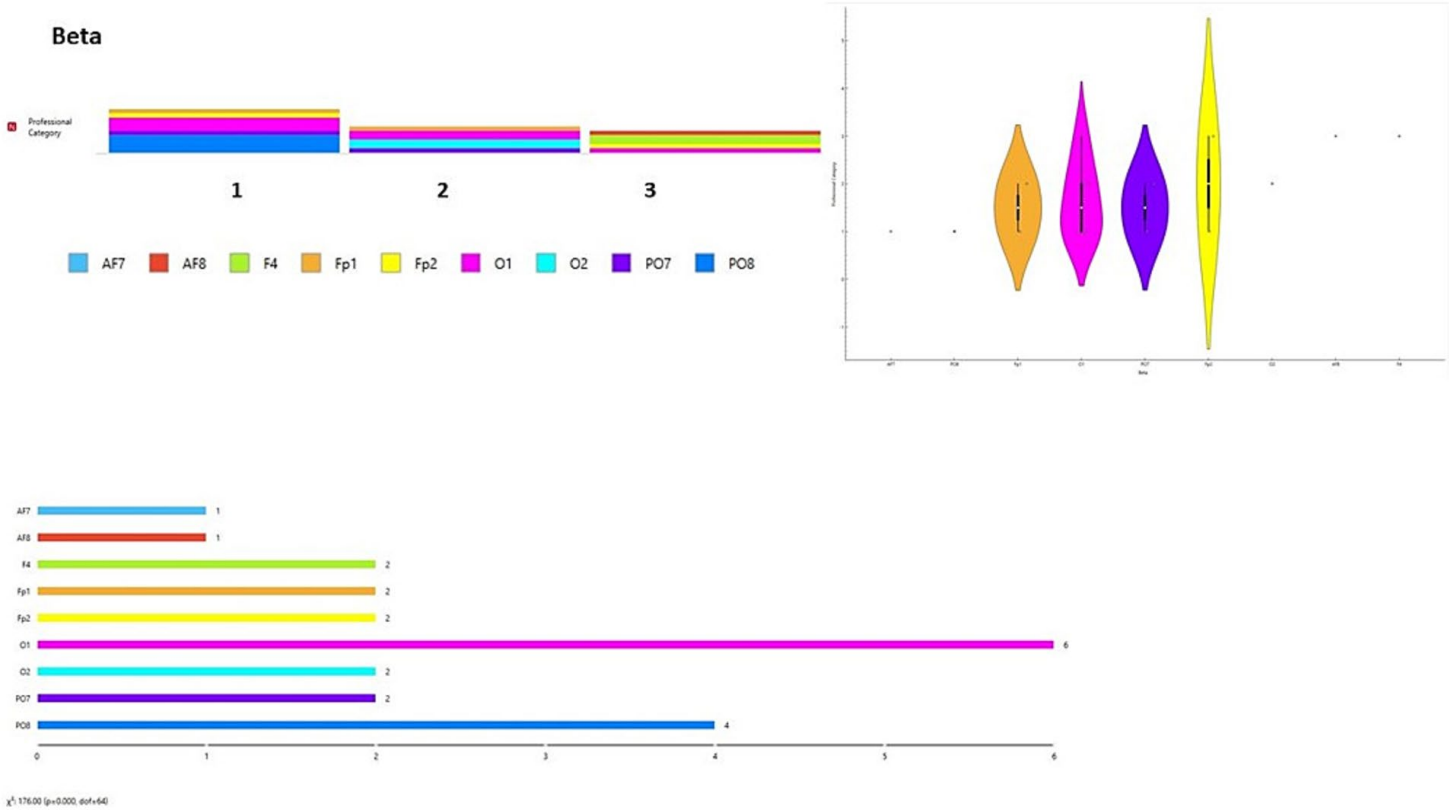
Beta wave analysis of the distribution of frequency of use in brain channels and Chi-square test (χ^2^). 1 = Group 1: final-year students; Group 2: junior lecturers; Group 3: senior lecturers.

**Figure 10:**
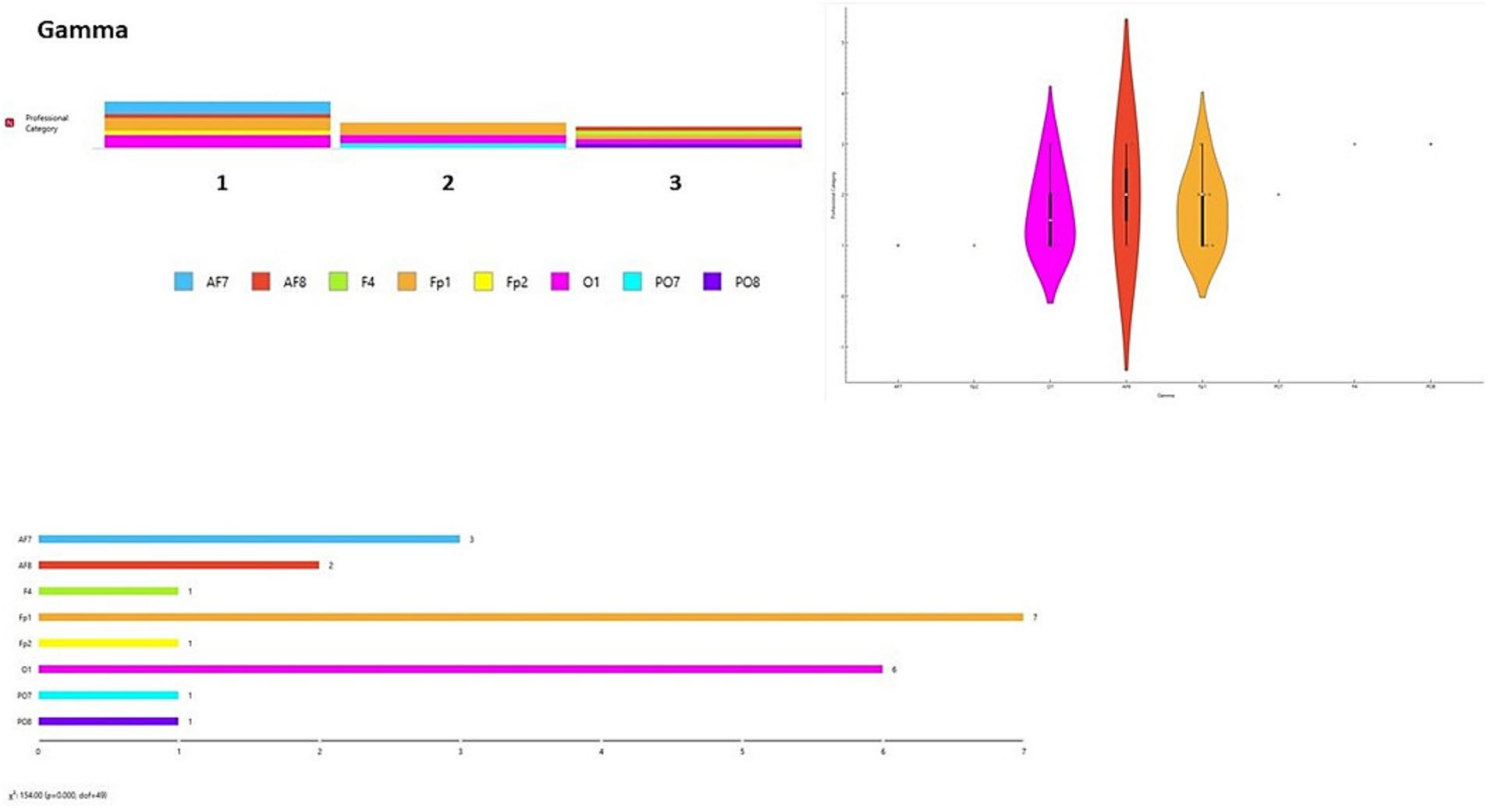
Gamma wave analysis of the distribution of frequency of use in brain channels and Chi-square test (χ^2^). 1 = Group 1: final-year students; Group 2: junior lecturers; Group 3: senior lecturers.

A data visualization analysis was also performed using Orange software. The distribution of channels was examined for each brainwave band in relation to participant type (final-year students, junior lecturers, senior lecturers). Chi-square tests (χ^2^) were applied to assess differences between observed and expected frequencies. Significant differences were found across all brainwave bands according to participant type [Delta wave χ^2^ = 154.00, *p* < 0.001 (Figure 6); Theta wave χ^2^ = 110.00, *p* < 0.001 (Figure 7); Alpha wave χ^2^ = 132.00, *p* < 0.001 (Figure 8); Beta wave χ^2^ = 176.00, *p* < 0.001 (Figure 9); Gamma wave χ^2^ = 154.00, *p* < 0.001 (Figure 10)].

These results are presented as descriptive patterns in spectral power, without implying direct functional or cognitive assignments to specific electrode locations.

The power of the EEG signals was calculated across five frequency bands: Delta (1–4 Hz), Theta (4–8 Hz), Alpha (8–13 Hz), Beta (13–30 Hz), and low Gamma (30–40 Hz). Power was obtained using the power spectral density via Welch’s method and is reported as the absolute average per channel across the entire recording for each participant. These data allow the characterization of brain activity across different regions (frontal, parietal, and occipital) and enable observation of the distribution of spectral power between participants. Overall, the results reflect the expected variability across channels and participants, showing patterns consistent with previous findings in resting-state EEG (consulted in Supplementary Table S4). Also, the analysis scripts are attached in the same document. Likewise, the code applied can be verified in Supplementary Figure S1 section.

## 4 Discussion

No significant differences were found between groups in perceived cognitive workload across the six dimensions [Group 1: final-year students; Group 2: adjunct lecturers; Group 3: full professors]. Similarly, no differences were observed between groups in the weighted NASA-TLX workload applied after the task of viewing an avatar representing different emotions. This indicates that all participants perceived a similar level of task difficulty in terms of cognitive workload indicators (mental demand, physical demand, temporal demand, effort, performance, frustration, and weighted workload), which is consistent with previous studies highlighting the inherent subjectivity of offline cognitive workload measurements (Veenman et al., 2014; Sweller, 2023).

Nevertheless, trend analyses revealed a moderate effect of the age factor on physical demand, effort, performance, frustration, and weighted workload, suggesting that age may influence the perception of certain components of cognitive workload.

Likewise, no significant differences were found between the three participant groups in maximum EEG power (μV^2^) recorded in the Delta (1–4 Hz), Theta (4–8 Hz), Alpha (8–13 Hz), Beta (13–30 Hz), and Gamma (>30 Hz) bands, although moderate effect sizes were observed for the Delta and Beta bands. In contrast, significant differences were detected for the gender factor in the Delta, Theta, Alpha, and Beta bands, with effect sizes ranging from moderate to large. These findings are consistent with the literature reporting gender-related differences in oscillatory brain activity, particularly in frequency bands associated with attention, cognitive control, and emotional processing (Knyazev, 2007; Babiloni et al., 2006; Güntekin and Başar, 2014).

In addition, participants were not evenly distributed across clusters. A more consistent correspondence was observed in Group 1 (final-year students) and Group 2 (adjunct lecturers), whereas greater dispersion was found in Group 3 (senior university professors with more than 15 years of experience). These results were confirmed by the frequency analysis. Specifically, the channel with the highest percentage of activation in the Delta band was Fp1 across all three groups, in line with previous studies on frontal activation during cognitive and emotional visualization tasks (Pesonen et al., 2007; Li et al., 2017; Nguyen et al., 2020; Spüler et al., 2016). Maximum Theta power was observed at Fp1 in Groups 1 and 2, and at F4 in Group 3, which is consistent with findings indicating that Theta activity is a robust predictor of individual differences in cognitive functioning (Grunwald et al., 2014; Roux and Uhlhaas, 2014; Spüler et al., 2016). For the Alpha, Beta, and Gamma bands, no consistent activation channels were observed across groups.

The absence of significant group differences in maximum EEG power, alongside the emergence of differences in frequency-based analyses, suggests that descriptive analyses should complement inferential approaches in this type of study in order to adequately interpret activation patterns and enable personalized responses for each participant or group (Sweller, 2023; Jamil et al., 2021). The greatest variability was detected in Group 3, which included participants with a wide range of ages, genders, professional categories, and years of experience (>15). Other individual factors, such as behavioral and affective aspects and device type, may also influence the results (Chella et al., 2016; Ignatiadis et al., 2022; Opitz et al., 2021; Jamil et al., 2021). Furthermore, variability in electrode placement, connectivity, and anatomical correspondence may limit the strictly localized interpretation of brain activity (Scrivener and Reader, 2022). Finally, as this is a pilot study with a small, non-random sample and task-specific stimulus images, all findings should be considered exploratory and interpreted with appropriate caution.

## 5 Conclusion

In sum, the use of EEG devices provides preliminary insights into cognitive processing during the performance of various tasks (Beauchemin et al., 2024). Specifically, EEG interfaces enable more direct and empirical monitoring of students’ cognitive states in relation to the cognitive load they actually experience (Spüler et al., 2016). However, multiple factors—including device type, participant characteristics (e.g., age, gender, professional status, emotional state, cognitive level, cranial structure)—may influence the recordings. Furthermore, accurate interpretation requires careful data processing. Studies should combine statistical and machine learning analyses with more individualized approaches, such as case studies, to fully contextualize the results.

This study found no significant differences in participants’ perceptions of the emotion visualization task across groups (final-year students, adjunct lecturers, full professors). Similarly, no significant differences were observed in power (μV^2^) across the frequency bands—Delta (1–4 Hz), Theta (4–8 Hz), Alpha (8–13 Hz), Beta (13–30 Hz), and Gamma (>30 Hz). Nonetheless, trends suggested potential differences related to factor age. However, significant differences were found with regard to gender in the Delta, Theta, Alpha, and Beta bands. These differences may be partly related to gender-specific variations in emotional processing elicited by the visual stimuli. Previous EEG research has shown that oscillatory activity in the Theta and Alpha bands is not only associated with cognitive demands such as attention, executive control, and working memory, but is also highly sensitive to the emotional content of visual information (Knyazev, 2007; Güntekin and Başar, 2014). Emotional processing has been linked to increased Theta power, reflecting integrative and evaluative processes, as well as to Alpha desynchronization, particularly in frontal and posterior regions, indicating enhanced attentional allocation to emotionally salient stimuli (Klimesch, 2012; Uusberg et al., 2014).

In this context, the observed gender-related differences in EEG power may reflect differences in affective engagement, emotional reactivity, or attentional strategies during the task, rather than purely cognitive load differences. This interpretation is consistent with previous studies reporting gender-related variability in oscillatory brain activity during tasks involving emotional perception and regulation (Knyazev, 2007; Babiloni et al., 2006; Güntekin and Başar, 2014). Nevertheless, these findings should be interpreted with caution due to the characteristics of the sample, which was based on a convenience and non-randomized selection. Future studies employing larger, randomly selected samples and alternative experimental designs will be necessary to further examine and validate the role of gender-related emotional and cognitive factors in modulating EEG activity.

Additionally, previously unlabeled groups were identified, with a contingency index of *C* = 0.43 between these groups and participant categories (final-year students, assistant professors, tenured professors). Key findings included:

Group 1: Coincidence in Theta activity (associated with memory and emotions) in the Fp1 channel (linked to attention and self-control), primarily related to participant groups 1 and 3.

Group 2: Correspondences in Delta, Theta, Alpha, and Beta activity (associated with attention, filtering, alertness, and emotions) in the F4 channel (related to emotional and social processing), linked to groups 1 and 3. Increased activity was also observed in the O1 channel (right-side visual processing). Additionally, Gamma activity (sensory integration) in the PO8 channel (rapid visual perception) corresponded to Group 2.

These results should be interpreted with caution due to the small, non-random sample. Nevertheless, these exploratory pilot studies provide valuable insights for designing future research and advancing neuropsychology, particularly through the practical application of EEG recordings in higher education learning contexts.

The implications of these findings for educational practice, potential future research directions, and study limitations are discussed below.

### 5.1 Application of the results to the higher education environment

The objective assessment of cognitive load during task performance represents a key opportunity for advancing personalized learning in higher education (Beauchemin et al., 2024; Dan and Reiner, 2017; Sarailoo et al., 2022). EEG-based measurements provide a promising means of capturing learners’ cognitive and affective states continuously and unobtrusively, which may support the design of adaptive learning environments that are more responsive to individual needs. In this regard, EEG devices have the potential to inform the optimization of students’ cognitive and metacognitive strategies during learning activities, fostering attention to learner diversity and enhancing educational outcomes.

Moreover, EEG-based brain–computer interface (BCI) systems could enable the implementation of neurofeedback approaches, allowing real-time adjustments to task difficulty, pacing, or instructional support based on learners’ cognitive load. However, the effective integration of these technologies into higher education settings requires careful consideration of signal processing, data interpretation, pedagogical alignment, and ethical implications, as well as ongoing improvements in device accuracy, reliability, and usability to ensure their feasibility in natural learning environments.

### 5.2 Limitations of the study

Several limitations of the present study should be acknowledged. First, the use of convenience sampling and the relatively small sample size (*n* = 22) limit both statistical power and the generalizability of the findings. Nevertheless, it is important to emphasize that the collection and processing of EEG data are inherently complex and time-consuming, as individual recording sessions lasted between 30 and 60 min per participant and required specialized computational expertise for signal extraction, cleaning, and analysis.

Second, as highlighted in previous research (Ignatiadis et al., 2022; Scrivener and Reader, 2022), the direct attribution of specific cognitive functions to individual electrodes within the 10–20 system represents a simplification. Anatomical variability, electrode placement accuracy, and the choice of reference can all influence the interpretation of EEG signals. In line with the recommendations of Opitz et al. (2021), this study adopted a descriptive analytical approach supported by frequency-based analyses, with the goal of generating hypotheses rather than drawing confirmatory conclusions.

Third, the study focused on a highly specific experimental task: the visualization of images of an avatar representing different emotional states. As such, caution should be exercised in generalizing the findings to other tasks or populations.

Despite these limitations, exploratory pilot studies of this kind remain highly valuable. They provide initial insights that can serve as a foundation for the development of more robust and targeted research designs, helping to refine hypotheses and guide subsequent studies in this field, even at early stages of investigation.

### 5.3 Future lines of research

Future research will build upon the present study by analyzing EEG markers in relation to the specific cognitive processing demands elicited by each stimulus, rather than focusing solely on peak power within each frequency band. Participant characteristics—including age, academic discipline, gender, cognitive ability, and cranial morphology—will also be systematically considered.

Moreover, subsequent studies will investigate participants’ responses to more complex stimuli relevant to higher education learning tasks. Increasing the sample size will be a key priority, as larger datasets will enable more robust applications of both supervised and unsupervised machine learning techniques and allow for more reliable assessment of cluster stability.

In addition, future research will incorporate three-dimensional brain localization methods, consistent with contemporary neuroscientific approaches that emphasize dynamic and individualized analyses (Chella et al., 2016; Opitz et al., 2021), including the MNE 3D visualization framework.

Finally, multimodal approaches integrating EEG with eye-tracking and galvanic skin response (GSR) data will be explored. Advanced signal processing techniques—such as time–frequency analysis, cross-band interaction metrics, and signal complexity measures (e.g., entropy, fractal dimension, and Lyapunov exponents)—will be applied to capture interactions that may not be evident in single-band analyses.

## Data Availability

The raw data supporting the conclusions of this article will be made available by the authors, without undue reservation.
