# Supplementary material for: Processing and analysis of portable EEG data for cognitive load assessment in neurotypical university students
Source: Front Hum Neurosci. 2026 Mar 19;20:1737723. doi: 10.3389/fnhum.2026.1737723 (PMC13044042; doi:10.3389/fnhum.2026.1737723)
Supplement: Supplementary file 1 [file Data_Sheet_1.docx]

Supplementary Material

**TABLE S1.** Relationship between brain areas and electrodes, and their association with the information processing analysis (authors’ own work).

| **Brain areas** | **Electrodes** | **Information processing** |
| --- | --- | --- |
| **Frontal and prefrontal**  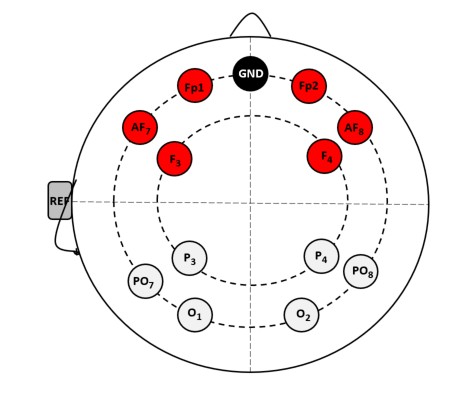 | Fp1 = front left pole  Fp2 = right front pole  AF7 = front anterior  AF8 = front anterior  F3 = Front  F4 = Front | Reference to reasoning, abstract thinking, self-control, decision making,  planning and pragmatic skills. Specifically:   \| Electrode \| Information processing meaning \| \| --- \| --- \| \| Fp1 \| Attention processes, self-control, executive functions, and also emotional processing. \| \| Fp2 \| Emotional assessment and rapid responses \| \| Processes related to anticipatory attention, planning, analysis of novel stimuli, and impulse control. \| \| \| AF7 \| Verbal planning \| \| AF8 \| Spatial and emotional planning \| \| Frontal lobes, but slightly behind Fp1/Fp2. High involvement in executive functions (such as problem solving, working memory, response inhibition). \| \| \| F3 \| More analytical, rational and verbal language tasks \| \| F4 \| Emotional processing, response to social stimuli and cognitive flexibility \| |
| **Parietales and Occipitals**  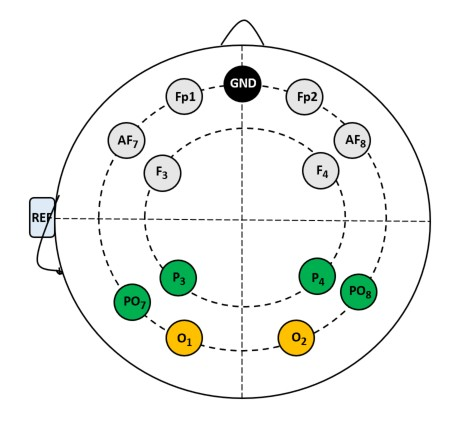 | P3 = Parietal  P4 = Parietal  PO7 = Parietal Occipital  PO8 = Parietal Occipital  O1 = Occipital  O2 = Occipital | Relation to somatic sensitivity (areas 5 and 7). The analysis and integration of sensory information in the inferior parietal lobe refers to complex perceptual experiences.   \| Electrode \| Information processing meaning \| \| --- \| --- \| \| P3 \| Logical and sequential spatial information processing \| \| P4 \| Comprehensive and global visual attention \| \| PO7 \| Detail-based spatial orientation \| \| PO8 \| Fast visual pattern processing and global perception \| \| O1 \| Processing visual details of the right field of vision \| \| O2 \| Processing visual details of the left field of vision \| |

**TABLE S2.** Contingency table showing the relationship between professional category membership and assigned cluster.

| **Cluster** | **Group** | **Wave** | **Channel** | **Graphical representation of brain activity** |
| --- | --- | --- | --- | --- |
| **1** | **1** | Delta | AF7 | 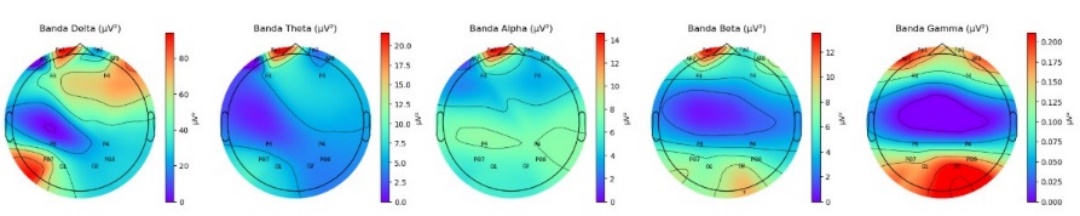 |
|  |  | **Theta** | **Fp1** |  |
|  |  | Alpha | PO7 |  |
|  |  | Beta | PO7 |  |
|  |  | Gamma | AF8 |  |
| **1** | **2** |  |  | - |
| **1** | **3** | Delta | Fp1 | 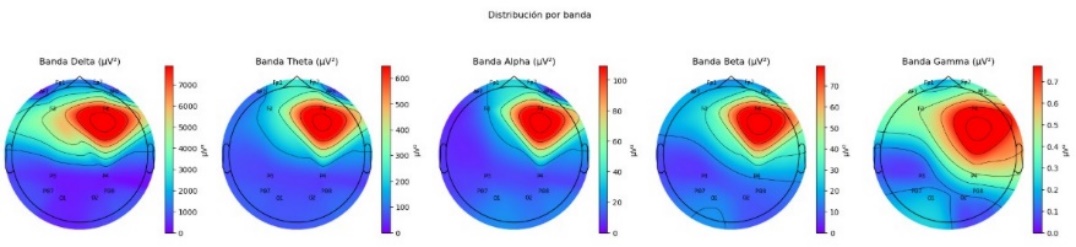 |
|  |  | **Theta** | **Fp1** |  |
|  |  | Alpha | Fp1 |  |
|  |  | Beta | PO8 |  |
|  |  | Gamma | O1 |  |
| **2** | **1** | **Delta** | **Fp1** | 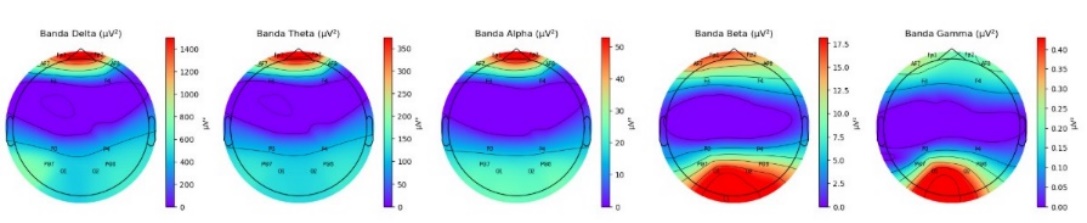 |
|  |  | **Theta** | **Fp1** |  |
|  |  | Alpha | Fp2 |  |
|  |  | Beta | O1 |  |
|  |  | Gamma | O1 |  |
| **2** | **2** |  |  | **-** |
| **2** | **3** | **Delta** | **Fp1** | 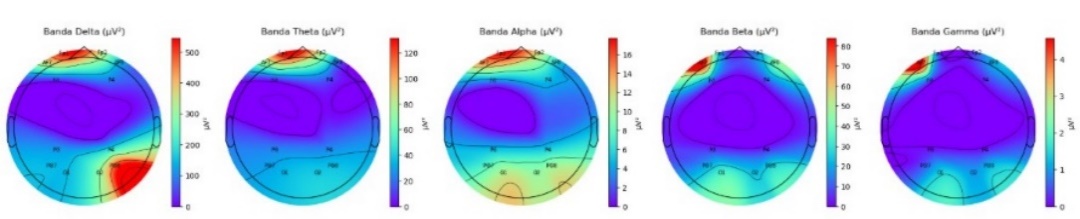 |
|  |  | **Theta** | **Fp1** |  |
|  |  | Alpha | Fp1 |  |
|  |  | Beta | AF7 |  |
|  |  | Gamma | AF7 |  |
| **3** | **1** | **Delta** | **F4** | 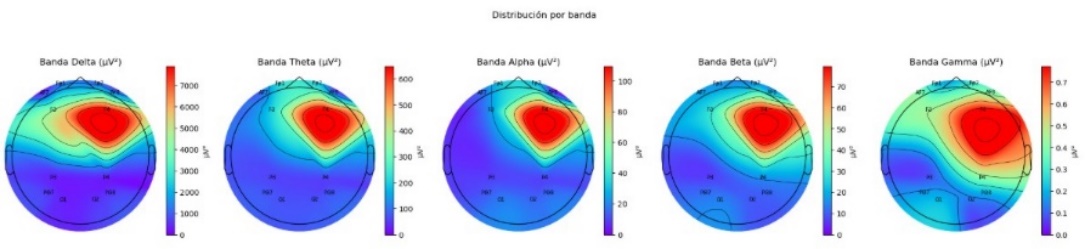 |
|  |  | **Theta** | **F4** |  |
|  |  | **Alpha** | **F4** |  |
|  |  | **Beta** | **F4** |  |
|  |  | Gamma | F4 |  |
| **3** | **2** | **Delta** | **F4** | 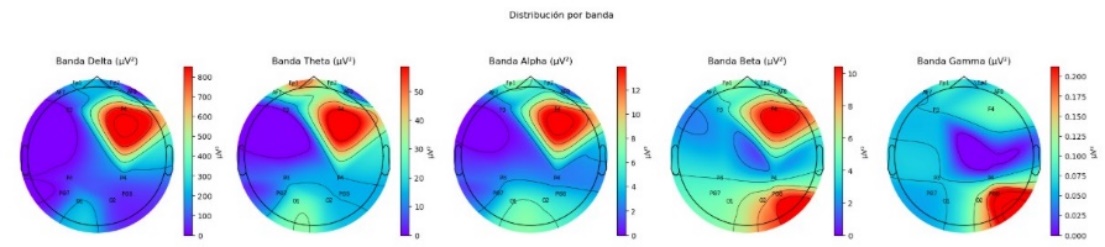 |
|  |  | **Theta** | **F4** |  |
|  |  | **Alpha** | **F4** |  |
|  |  | **Beta** | **F4** |  |
|  |  | Gamma | PO8 |  |
| **3** | **3** | Delta | O1 | 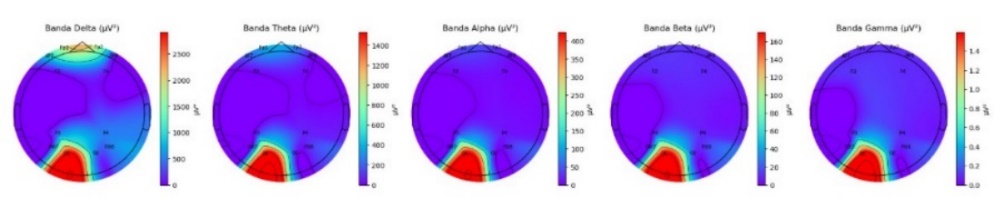 |
|  |  | Theta | O1 |  |
|  |  | Alpha | O1 |  |
|  |  | Beta | O1 |  |
|  |  | Gamma | O1 |  |

**TABLE S3.** Frequency and percentage analysis of brainwave activation across the different channels.

| **Group** | **Delta** | **Frequency** | **Total Delta** | **Theta** | **Frequency** | **% Total Theta** | **Alpha** | **Frequency** | **%**  **Total Alpha** | **Beta** | **Frequency** | **% Total Beta** | **Gamma** | **Frequency** | **%**  **Total Gamma** |
| --- | --- | --- | --- | --- | --- | --- | --- | --- | --- | --- | --- | --- | --- | --- | --- |
| 1 | AF7 | 9.1 | 4.5 | AF8 | 18.18 | 9.1 | **Fp1** | **36.4** | **36.4** | Fp1 | 18.2 | 13.63 | O2 | 9.1 | 4.5 |
| 1 | AF8 | 18.2 | 9.1 | **Fp1** | **54.54** | **54.54** | O1 | 18.2 | 13.63 | O1 | 18.2 | **22.72** | AF7 | 18.2 | 13.63 |
| 1 | **Fp1** | **45.45** | **50.0** | Fp2 | 9.1 | 13.6 | Fp2 | 18.2 | 22.72 | **PO8** | **36.36** | 18.18 | **Fp1** | **27.3** | **31.81** |
| 1 | Fp2 | 9.1 | 9.1 | PO8 | 18.18 | 4.5 | PO8 | 18.2 | 9.1 | Fp2 | 9.1 | 9.1 | Fp2 | 9.1 | 4.5 |
| 1 | PO8 | 18.2 | 9.1 |  |  |  | PO7 | 9.1 | 9.1 | O1 | 18.2 |  | **O1** | **27.3** | 22.7 |
| 1 |  |  |  |  |  |  |  |  |  | PO7 | 18.2 | 13.63 | AF8 | 9.1 | 9.1 |
| 2 | **Fp1** | **66.6** |  | **Fp1** | **66.66** |  | AF7 | 16.66 |  | AF7 | 16.66 | 4.5 | AF7 | 16.66 |  |
| 2 | Fp2 | 16.6 |  | Fp2 | 33.33 |  | O1 | 16.66 |  | O1 | 16.66 |  | O1 | 16.66 |  |
| 2 | PO7 | 16.6 | 4.5 |  |  |  | **O2** | **33.33** |  | **O2** | **33.33** | 9.1 | **Fp1** | **50** |  |
| 2 |  |  |  |  |  |  | Fp1 | 16.66 |  | Fp1 | 16.66 |  | PO7 | 16.66 | 9.1 |
|  |  |  |  |  |  |  | PO7 | 16.66 |  | PO7 | 16.66 |  |  |  |  |
| 3 | O1 | 20 | 4.5 | O1 | 20 | 4.5 | **O1** | **20** |  | **O1** | **20** |  | **O1** | **20** |  |
| 3 | F4 | **40** | 9.1 | **F4** | **40** | 9.1 | **F4** | **20** | 9.1 | **F4** | **20** | 4.5 | **PO8** | **20** | 4.5 |
| 3 | **Fp1** | **40** |  | **Fp1** | **40** |  | **AF8** | **20** |  | **AF8** | **20** | 4.5 | **AF8** | **20** |  |
| 3 |  |  |  |  |  |  | **Fp2** | **20** |  | **Fp2** | **20** | 9.1 | **Fp1** | **20** |  |
| 3 |  |  |  |  |  |  | **PO7** | **20** |  | **PO7** | **20** |  | **PO7** | **20** |  |

Note: % = Percentages: 4.5-19%, 20-39%, 40%-70%; Group: Group 1: final-year students; Group 2: junior lecturers; Group 3: senior lecturers.

**TABLE S4.** Raw channel power values.

| **Subject** | **Channel** | **Delta** | **Theta** | **Alpha** | **Beta** | **Gamma low** |
| --- | --- | --- | --- | --- | --- | --- |
| 1 | AF7 | 28.0699727 | 6.722073097 | 5.347519641 | 12.35991021 | 5.471648348 |
| 1 | Fp1 | 24.56000968 | 6.274856273 | 4.935953523 | 10.24137038 | 4.868522266 |
| 1 | Fp2 | 22.8498513 | 6.239211737 | 5.233056056 | 9.716046896 | 4.793250811 |
| 1 | AF8 | 45.22360641 | 7.019477584 | 6.523207452 | 13.55271853 | 6.801457391 |
| 1 | F3 | 86.86225187 | 10.58395873 | 7.742567323 | 12.90767727 | 6.287138359 |
| 1 | F4 | 110.1890597 | 11.27624094 | 9.088840255 | 15.34301713 | 8.027576833 |
| 1 | P3 | 31.71382829 | 9.158457735 | 18.02138213 | 16.07713036 | 7.705331713 |
| 1 | P4 | 43.33306896 | 12.65570599 | 19.80850001 | 16.90617291 | 7.422277737 |
| 1 | PO7 | 52.11775359 | 9.676853997 | 10.0095174 | 15.88323647 | 8.382848599 |
| 1 | O1 | 35.44428047 | 8.274440002 | 12.6635424 | 19.38341297 | 9.399709586 |
| 1 | O2 | 26.80669963 | 7.826046251 | 14.08219933 | 21.93558941 | 11.27674846 |
| 1 | PO8 | 36.31570861 | 9.156392313 | 16.45816577 | 19.85132863 | 10.56790924 |
| 2 | AF7 | 532.7162385 | 111.0783733 | 25.74908035 | 30.00077955 | 5.766985208 |
| 2 | Fp1 | 531.8974983 | 112.2937551 | 26.4867023 | 28.5015499 | 5.074564266 |
| 2 | Fp2 | 495.9234968 | 109.2540519 | 24.32229241 | 21.80326639 | 3.51414129 |
| 2 | AF8 | 472.5476076 | 105.6051332 | 20.48925308 | 20.46128384 | 4.592215605 |
| 2 | F3 | 463.5211783 | 112.9123618 | 27.47505023 | 28.18182263 | 6.967736648 |
| 2 | F4 | 522.534013 | 116.5393603 | 26.13961505 | 25.8902611 | 5.584044856 |
| 2 | P3 | 442.8874368 | 104.4281277 | 21.66685661 | 18.74150056 | 3.33568944 |
| 2 | P4 | 440.8267603 | 108.1180829 | 21.73082382 | 22.10507344 | 4.06067157 |
| 2 | PO7 | 425.1443702 | 111.954769 | 23.94899648 | 21.9010883 | 5.352341999 |
| 2 | O1 | 417.8520116 | 112.5253638 | 26.86519259 | 42.14268294 | 5.677408796 |
| 2 | O2 | 410.3431095 | 115.7305939 | 24.91311216 | 24.61036735 | 5.758953326 |
| 2 | PO8 | 444.4230078 | 111.8368228 | 25.41292925 | 26.07193839 | 5.437286463 |
| 3 | AF7 | 99.49198701 | 13.46120984 | 7.780271352 | 47.37236036 | 22.26200863 |
| 3 | Fp1 | 90.38890194 | 13.84380415 | 5.756201096 | 12.84630559 | 6.344247342 |
| 3 | Fp2 | 85.11403164 | 12.01164364 | 5.656318775 | 15.87465387 | 7.359010754 |
| 3 | AF8 | 100.2975538 | 14.64506222 | 8.761944259 | 29.38247124 | 13.85142021 |
| 3 | F3 | 104.9952985 | 16.16897462 | 7.295705673 | 14.64083066 | 6.555562111 |
| 3 | F4 | 1152.628131 | 104.0498505 | 29.38018324 | 28.94445996 | 8.004575185 |
| 3 | P3 | 101.1776891 | 15.17487793 | 10.68858569 | 18.82573289 | 9.455529975 |
| 3 | P4 | 159.7997632 | 17.07397719 | 12.41114748 | 17.77672018 | 8.87324332 |
| 3 | PO7 | 86.45930831 | 12.20484382 | 7.068289866 | 20.52190175 | 11.25375403 |
| 3 | O1 | 85.19303111 | 13.66677164 | 7.706746746 | 20.05319059 | 10.24833891 |
| 3 | O2 | 302.1350359 | 22.20644984 | 14.6063803 | 47.38460868 | 32.53722224 |
| 3 | PO8 | 248.2406078 | 21.39614384 | 15.3471263 | 45.42602435 | 29.61689025 |
| 4 | AF7 | 867.4678903 | 148.6776491 | 18.92598763 | 16.03492609 | 6.189299953 |
| 4 | Fp1 | 860.0839962 | 155.5838967 | 20.94388785 | 10.95239718 | 3.366475442 |
| 4 | Fp2 | 863.8145171 | 156.1216472 | 20.99955359 | 11.62271124 | 3.569818362 |
| 4 | AF8 | 859.8578982 | 153.2593101 | 20.3941432 | 11.68117214 | 3.853275546 |
| 4 | F3 | 2549.427687 | 222.0312383 | 26.89920157 | 16.17308218 | 4.059962204 |
| 4 | F4 | 871.4604329 | 168.188443 | 24.5971032 | 15.22606832 | 4.578674944 |
| 4 | P3 | 876.0023576 | 157.1908109 | 23.33569842 | 13.32077861 | 4.046779737 |
| 4 | P4 | 877.9651316 | 162.395112 | 25.30164428 | 15.18304053 | 5.137942011 |
| 4 | PO7 | 1089.767673 | 168.681866 | 24.15413881 | 15.96937978 | 5.633819734 |
| 4 | O1 | 850.2093748 | 157.1708706 | 27.95737303 | 23.20325981 | 8.167197146 |
| 4 | O2 | 884.9681286 | 158.9128573 | 31.28621899 | 37.08186565 | 15.9796062 |
| 4 | PO8 | 984.6107076 | 164.4544837 | 30.19366663 | 30.77801033 | 12.98148336 |
| 5 | AF7 | 516.8122294 | 89.94561507 | 17.25566532 | 15.19475087 | 3.732516391 |
| 5 | Fp1 | 532.7025016 | 102.8324433 | 21.52484368 | 17.3658821 | 4.035692566 |
| 5 | Fp2 | 467.1736445 | 65.1981406 | 13.00246525 | 11.59276018 | 2.905078252 |
| 5 | AF8 | 477.4554205 | 66.21090404 | 11.16808462 | 10.56009471 | 3.019286385 |
| 5 | F3 | 471.6440637 | 67.02482632 | 15.92343521 | 17.27416613 | 3.872041074 |
| 5 | F4 | 472.73539 | 66.00231132 | 15.308011 | 18.38836705 | 3.860650059 |
| 5 | P3 | 479.572026 | 63.82322398 | 20.26910629 | 21.44357113 | 3.588638676 |
| 5 | P4 | 620.4995757 | 57.77917576 | 19.1913648 | 20.87825963 | 3.631541853 |
| 5 | PO7 | 455.5991252 | 61.03247686 | 18.19604032 | 21.82636675 | 3.937582115 |
| 5 | O1 | 2942.910355 | 1636.267258 | 475.3566506 | 201.266499 | 21.16197616 |
| 5 | O2 | 478.0271284 | 65.55955204 | 23.83650977 | 27.02194987 | 5.205670011 |
| 5 | PO8 | 500.2833272 | 68.35693341 | 23.12012663 | 23.55399356 | 4.449681655 |
| 6 | AF7 | 196.1043699 | 57.85611034 | 12.52709248 | 8.536801068 | 2.440454817 |
| 6 | Fp1 | 184.9271179 | 50.17649591 | 11.45978903 | 7.127242413 | 1.733868699 |
| 6 | Fp2 | 175.5913293 | 46.84551612 | 10.77719152 | 5.764080975 | 1.3268167 |
| 6 | AF8 | 180.3179049 | 49.71310603 | 10.91859762 | 6.249386527 | 1.557336365 |
| 6 | F3 | 231.0276795 | 47.32514833 | 12.04353113 | 8.550085698 | 1.783568694 |
| 6 | F4 | 186.6458852 | 49.37743344 | 12.57177978 | 8.612020803 | 1.928167765 |
| 6 | P3 | 179.4157517 | 48.95945623 | 14.42491992 | 10.34304022 | 1.891754189 |
| 6 | P4 | 180.9107927 | 53.0159305 | 15.82055859 | 10.42334183 | 1.636183694 |
| 6 | PO7 | 179.4856597 | 48.51317864 | 12.57509002 | 7.334545302 | 1.366919204 |
| 6 | O1 | 180.8500218 | 53.04647304 | 15.55552038 | 12.38435119 | 2.224309412 |
| 6 | O2 | 232.048342 | 76.45294042 | 29.53819871 | 28.72418831 | 4.748842469 |
| 6 | PO8 | 181.5382553 | 53.43816527 | 18.08468816 | 12.77108504 | 2.183608986 |
| 7 | AF7 | 270.0966598 | 33.06386007 | 9.206840478 | 26.95724042 | 8.433259542 |
| 7 | Fp1 | 294.4562312 | 42.56267347 | 13.54782584 | 40.12482083 | 14.05131743 |
| 7 | Fp2 | 260.5192853 | 40.24537743 | 11.13814378 | 14.20584829 | 3.273769986 |
| 7 | AF8 | 259.3700718 | 33.9133522 | 9.340001511 | 17.51352046 | 4.744645485 |
| 7 | F3 | 264.6058549 | 43.23340398 | 11.42692308 | 16.50167743 | 4.423592259 |
| 7 | F4 | 263.0984688 | 41.47996598 | 10.12416523 | 13.85305782 | 3.551398056 |
| 7 | P3 | 236.5376193 | 45.15512201 | 11.63231927 | 16.71211632 | 4.413648908 |
| 7 | P4 | 241.5898375 | 43.33803278 | 12.31454091 | 17.06821117 | 4.487528706 |
| 7 | PO7 | 255.2709313 | 65.94919173 | 18.51913952 | 23.45236319 | 6.510394943 |
| 7 | O1 | 1191.522679 | 156.206336 | 67.64827012 | 29.76663027 | 5.953456148 |
| 7 | O2 | 402.7201137 | 70.77625175 | 23.42690083 | 26.52595602 | 6.511892139 |
| 7 | PO8 | 2150.961449 | 252.4113301 | 68.78579613 | 34.73958829 | 8.128232311 |
| 8 | AF7 | 421.5683748 | 50.5981086 | 9.200191905 | 10.65693582 | 2.564372264 |
| 8 | Fp1 | 460.0242183 | 56.10928827 | 12.43007115 | 22.57365123 | 5.787717893 |
| 8 | Fp2 | 470.369796 | 58.10997313 | 14.61022861 | 33.05188578 | 10.96947546 |
| 8 | AF8 | 448.3643198 | 54.7072021 | 12.68886452 | 28.33661497 | 10.56923876 |
| 8 | F3 | 415.4901228 | 56.36968932 | 13.21494526 | 18.92450339 | 4.447484078 |
| 8 | F4 | 452.3840452 | 56.62613487 | 13.29088919 | 18.15460217 | 3.885284274 |
| 8 | P3 | 423.5463065 | 52.74314308 | 9.977640681 | 14.53790378 | 3.168751535 |
| 8 | P4 | 434.4437577 | 52.74353608 | 10.60357719 | 14.85950823 | 3.429687896 |
| 8 | PO7 | 411.7762484 | 53.37100865 | 8.864645076 | 14.12005431 | 3.47432039 |
| 8 | O1 | 469.2324774 | 57.25936262 | 10.94394844 | 14.41166916 | 3.609002286 |
| 8 | O2 | 408.552348 | 52.23631815 | 9.641337073 | 14.5493957 | 3.5752225 |
| 8 | PO8 | 449.920498 | 52.60909569 | 10.56718439 | 14.77537859 | 3.564973395 |
| 9 | AF7 | 918.8698893 | 180.1254887 | 25.13070984 | 13.67499936 | 2.742370185 |
| 9 | Fp1 | 1815.880751 | 195.5485362 | 37.56950758 | 31.31000993 | 6.970223906 |
| 9 | Fp2 | 1081.24161 | 167.4070226 | 21.712122 | 11.72375448 | 2.565628622 |
| 9 | AF8 | 1582.913434 | 183.7225498 | 29.97666594 | 21.08861678 | 4.65546403 |
| 9 | F3 | 892.0094891 | 186.151691 | 27.13343565 | 15.16802762 | 3.239514261 |
| 9 | F4 | 897.767643 | 181.7685575 | 23.96423376 | 12.30645142 | 2.45110672 |
| 9 | P3 | 834.8995877 | 196.4312077 | 27.90523072 | 13.78050007 | 2.89517418 |
| 9 | P4 | 862.0075806 | 197.9040779 | 31.52983376 | 17.67846486 | 3.267423667 |
| 9 | PO7 | 1174.581575 | 170.2364626 | 29.37169981 | 14.19276466 | 3.1745322 |
| 9 | O1 | 927.1345323 | 194.4997223 | 29.88672887 | 16.85957791 | 3.484540864 |
| 9 | O2 | 850.8111899 | 211.5015866 | 42.43581709 | 26.93054297 | 5.253079566 |
| 9 | PO8 | 1018.064524 | 338.3670812 | 95.77996265 | 41.15910082 | 6.67040238 |
| 10 | AF7 | 689.9576923 | 159.3948401 | 32.93764579 | 16.26752263 | 3.81652991 |
| 10 | Fp1 | 691.0561584 | 159.7558611 | 35.39541131 | 14.95185454 | 2.355576052 |
| 10 | Fp2 | 740.8105299 | 160.2087598 | 35.68165225 | 31.21304137 | 18.22636516 |
| 10 | AF8 | 710.3245862 | 161.8086759 | 31.11416832 | 17.39787042 | 6.199180247 |
| 10 | F3 | 723.2587819 | 164.1197363 | 36.95352482 | 19.90820137 | 3.424204165 |
| 10 | F4 | 689.7927122 | 158.5269984 | 34.90299399 | 16.47278083 | 2.054252907 |
| 10 | P3 | 669.3672781 | 167.0606016 | 36.53260963 | 17.00258246 | 3.496250228 |
| 10 | P4 | 667.4865652 | 172.1877866 | 34.37235644 | 16.41133438 | 2.973759249 |
| 10 | PO7 | 674.6031926 | 182.9021013 | 37.84897849 | 16.55951648 | 3.458769661 |
| 10 | O1 | 642.7088116 | 189.9640228 | 39.69160736 | 19.74194855 | 4.069169742 |
| 10 | O2 | 643.7894794 | 193.3846652 | 37.99245058 | 19.74460361 | 4.028238238 |
| 10 | PO8 | 652.0066177 | 178.60422 | 35.46244764 | 18.76749378 | 4.042514105 |
| 11 | AF7 | 51.16683807 | 21.69569131 | 19.97915555 | 32.72901029 | 13.3360668 |
| 11 | Fp1 | 57.40573206 | 23.01403838 | 21.91362261 | 19.58764981 | 6.908684008 |
| 11 | Fp2 | 77.53691311 | 24.07500297 | 25.50210351 | 12.13431755 | 3.08251354 |
| 11 | AF8 | 87.75476794 | 23.32845925 | 23.98019959 | 15.80803737 | 5.707728783 |
| 11 | F3 | 88.35443935 | 29.41764544 | 29.66524217 | 15.19872318 | 3.679639952 |
| 11 | F4 | 85.60293975 | 30.40373176 | 35.06899458 | 15.33504371 | 3.150493108 |
| 11 | P3 | 78.58298913 | 25.04354588 | 29.72394662 | 11.93323221 | 2.904303715 |
| 11 | P4 | 67.8678644 | 25.03828454 | 29.16366913 | 12.17452099 | 2.763074975 |
| 11 | PO7 | 133.5856145 | 33.11339054 | 25.44147264 | 14.20808179 | 4.498797477 |
| 11 | O1 | 331.009866 | 114.6930312 | 73.65736572 | 50.96008137 | 8.82233194 |
| 11 | O2 | 73.57365018 | 26.69760352 | 26.36168764 | 13.53621365 | 3.145624147 |
| 11 | PO8 | 641.0371261 | 357.4237204 | 102.7492237 | 39.64898531 | 5.392048776 |
| 12 | AF7 | 8640.477418 | 529.1846629 | 65.79714983 | 34.54366419 | 14.48787606 |
| 12 | Fp1 | 1518.737026 | 190.3918407 | 40.53393062 | 67.88726965 | 34.34737021 |
| 12 | Fp2 | 798.6923407 | 170.3371663 | 27.66187149 | 24.18798656 | 9.236308965 |
| 12 | AF8 | 1023.79317 | 166.979095 | 25.70225722 | 48.44728996 | 23.04259094 |
| 12 | F3 | 765.7266803 | 151.4056069 | 27.44426379 | 39.83597088 | 17.65286072 |
| 12 | F4 | 745.7200207 | 176.1495895 | 31.33111041 | 26.56095956 | 8.518548144 |
| 12 | P3 | 717.0985121 | 182.6526882 | 35.31411368 | 38.17170811 | 14.71388961 |
| 12 | P4 | 715.6259543 | 175.9914202 | 36.34286887 | 31.35017987 | 11.21565975 |
| 12 | PO7 | 2052.414408 | 1040.704128 | 438.8511863 | 125.5566687 | 19.01719935 |
| 12 | O1 | 707.5642743 | 186.0103837 | 49.90363373 | 40.86909586 | 11.5911893 |
| 12 | O2 | 741.8584416 | 181.723814 | 56.43854059 | 37.33092458 | 10.83462754 |
| 12 | PO8 | 762.2697809 | 196.058615 | 81.04532511 | 49.03921337 | 17.02238639 |
| 13 | AF7 | 55.76952325 | 10.04927951 | 2.111800165 | 5.794281636 | 1.960015251 |
| 13 | Fp1 | 54.53973899 | 9.96955264 | 2.03405882 | 5.583698551 | 2.021882374 |
| 13 | Fp2 | 55.44865783 | 9.361225185 | 2.151280011 | 5.334498023 | 1.978074942 |
| 13 | AF8 | 59.78699644 | 11.39352646 | 2.391753241 | 6.165490407 | 2.277820409 |
| 13 | F3 | 62.32985735 | 11.71143236 | 3.018364498 | 8.312104257 | 2.343166669 |
| 13 | F4 | 1132.997921 | 71.15196188 | 16.07476332 | 16.66139647 | 3.204038214 |
| 13 | P3 | 59.64439855 | 11.93798651 | 3.380587724 | 8.233202264 | 2.64116806 |
| 13 | P4 | 117.5677363 | 12.46472865 | 3.600618796 | 8.712322779 | 3.233921977 |
| 13 | PO7 | 54.86294182 | 9.47382601 | 2.532066959 | 5.852540123 | 2.055729749 |
| 13 | O1 | 237.2293491 | 17.08589954 | 5.458907386 | 7.627629042 | 2.46063726 |
| 13 | O2 | 60.1635237 | 12.67310211 | 6.156902252 | 12.7315678 | 5.102219459 |
| 13 | PO8 | 59.7187943 | 11.49391163 | 5.214093234 | 14.14324466 | 6.827267261 |
| 14 | AF7 | 610.0377996 | 71.72190312 | 14.26898168 | 16.51371481 | 2.250359644 |
| 14 | Fp1 | 642.9618714 | 74.443318 | 17.74328711 | 20.74126325 | 2.491566842 |
| 14 | Fp2 | 611.4255646 | 71.82175368 | 15.23580439 | 17.8756938 | 2.367989449 |
| 14 | AF8 | 582.3306744 | 67.1076875 | 12.12400547 | 13.99722504 | 1.891813808 |
| 14 | F3 | 592.1648373 | 64.7135356 | 12.58350576 | 14.20231984 | 2.112755844 |
| 14 | F4 | 583.9338502 | 71.17217216 | 13.2459685 | 15.50714942 | 2.184773045 |
| 14 | P3 | 597.7724188 | 64.40237769 | 9.684630284 | 11.90460921 | 2.241955299 |
| 14 | P4 | 610.1618792 | 70.27616468 | 13.17842941 | 18.04306708 | 2.554581721 |
| 14 | PO7 | 630.0977839 | 68.89953349 | 16.65146612 | 25.10360259 | 5.202707969 |
| 14 | O1 | 609.643496 | 68.58246681 | 19.70930127 | 27.45731808 | 4.246100973 |
| 14 | O2 | 619.0193264 | 71.73232778 | 25.23807113 | 32.80303414 | 4.034423471 |
| 14 | PO8 | 610.7654831 | 67.32566462 | 18.93393945 | 26.76431759 | 3.394124347 |
| 15 | AF7 | 197.5580288 | 19.98393806 | 9.80111942 | 15.65258421 | 1.998980053 |
| 15 | Fp1 | 198.2028292 | 20.79477607 | 10.43328685 | 16.58095423 | 1.925701185 |
| 15 | Fp2 | 197.5007931 | 19.36336524 | 10.10071712 | 24.492271 | 5.437513182 |
| 15 | AF8 | 202.3872964 | 19.54699098 | 11.78882523 | 55.45773465 | 21.7684517 |
| 15 | F3 | 199.7953505 | 21.16742275 | 11.77264455 | 18.49528596 | 1.838825046 |
| 15 | F4 | 204.0721167 | 21.9927728 | 12.81619958 | 19.57212236 | 2.289690541 |
| 15 | P3 | 189.6111434 | 22.32339469 | 12.07993598 | 19.20324973 | 1.815600095 |
| 15 | P4 | 199.2749833 | 30.06798739 | 19.6523276 | 22.92930477 | 2.30017629 |
| 15 | PO7 | 182.7398998 | 23.33816825 | 11.80000894 | 17.32885245 | 2.739525225 |
| 15 | O1 | 215.0145746 | 27.56620496 | 13.66154708 | 19.2282211 | 2.699961491 |
| 15 | O2 | 184.2443113 | 25.14482073 | 13.20732545 | 18.65572153 | 2.069251063 |
| 15 | PO8 | 204.5202328 | 24.86408586 | 16.56584495 | 19.39871141 | 2.341355289 |
| 16 | AF7 | 206.3256033 | 48.8357191 | 15.27949691 | 29.21112814 | 12.69093676 |
| 16 | Fp1 | 241.5369846 | 58.78260523 | 26.64743218 | 87.78400941 | 43.59326759 |
| 16 | Fp2 | 169.1630058 | 43.58895347 | 14.07616846 | 26.50741978 | 12.30583737 |
| 16 | AF8 | 153.5582586 | 40.76242339 | 12.43335406 | 16.62239312 | 6.613038631 |
| 16 | F3 | 286.2763927 | 77.48744829 | 24.0080442 | 51.36798901 | 17.32021086 |
| 16 | F4 | 178.2316163 | 46.72836076 | 15.38864759 | 26.59690199 | 9.175979835 |
| 16 | P3 | 151.537491 | 40.49247179 | 13.08593932 | 15.82798314 | 5.355865461 |
| 16 | P4 | 157.865887 | 41.60674226 | 14.27462736 | 16.09263109 | 5.266017578 |
| 16 | PO7 | 56139.39677 | 14238.35099 | 3601.87666 | 1242.013691 | 70.10778106 |
| 16 | O1 | 148.0442259 | 39.57348606 | 13.95428253 | 24.1370292 | 9.301668178 |
| 16 | O2 | 272.962756 | 49.02643047 | 15.04675623 | 19.14306524 | 7.485998092 |
| 16 | PO8 | 153.6009303 | 41.42508735 | 12.96922181 | 16.80381384 | 6.675867285 |
| 17 | AF7 | 101.0445237 | 32.3154492 | 6.921994887 | 5.86530913 | 1.810069942 |
| 17 | Fp1 | 92.96257695 | 31.04779498 | 7.289061322 | 5.899996945 | 1.734906686 |
| 17 | Fp2 | 91.42271825 | 31.93428123 | 7.218826145 | 6.128077432 | 1.754259452 |
| 17 | AF8 | 94.65434532 | 31.23613974 | 7.365385701 | 6.280630105 | 1.923547 |
| 17 | F3 | 98.85183833 | 32.65372277 | 7.831192099 | 6.357997068 | 1.628800935 |
| 17 | F4 | 104.6282671 | 35.59375214 | 9.355631388 | 8.282093936 | 2.544144569 |
| 17 | P3 | 108.2749086 | 30.0999022 | 8.038205922 | 6.626070221 | 1.926131509 |
| 17 | P4 | 112.8866282 | 33.49373255 | 9.012513736 | 8.667553006 | 4.982347787 |
| 17 | PO7 | 244.4862727 | 38.03731134 | 10.25630336 | 12.93912678 | 7.033032211 |
| 17 | O1 | 166.0256548 | 44.94720517 | 12.80621895 | 10.45963384 | 2.596648185 |
| 17 | O2 | 99.17036236 | 35.26365053 | 10.61823403 | 8.301215312 | 1.976910543 |
| 17 | PO8 | 102.1090694 | 35.03176946 | 9.122945831 | 7.70590751 | 2.158578119 |
| 18 | AF7 | 851.2394837 | 177.4686612 | 28.2296381 | 28.85704753 | 10.33579965 |
| 18 | Fp1 | 877.6131136 | 189.2884546 | 35.6077764 | 43.31672566 | 14.45682635 |
| 18 | Fp2 | 1103.43175 | 202.2107703 | 42.25412476 | 49.65786446 | 14.07623839 |
| 18 | AF8 | 814.7724074 | 184.8516793 | 30.85450227 | 29.84617857 | 7.380143539 |
| 18 | F3 | 949.9972714 | 219.5012701 | 45.98762718 | 27.02270834 | 6.970036876 |
| 18 | F4 | 57360.6351 | 2022.114774 | 197.445183 | 54.83648889 | 9.65567187 |
| 18 | P3 | 842.5803432 | 187.1231436 | 35.95225632 | 32.13307317 | 4.060342529 |
| 18 | P4 | 785.738614 | 191.8881435 | 36.62966144 | 26.18652945 | 3.835761673 |
| 18 | PO7 | 780.3259104 | 181.6937878 | 33.02041228 | 33.16039356 | 6.337024219 |
| 18 | O1 | 3311.10645 | 220.283244 | 40.77847224 | 35.58850337 | 7.246092824 |
| 18 | O2 | 817.4199881 | 198.1968888 | 38.09791982 | 31.78633154 | 7.691583769 |
| 18 | PO8 | 890.0281912 | 201.3199297 | 39.97048166 | 31.68834815 | 7.285707601 |
| 19 | AF7 | 581.0420847 | 73.07189237 | 14.2694283 | 18.08988952 | 12.35289963 |
| 19 | Fp1 | 471.5361555 | 72.64903237 | 14.22446851 | 13.70791168 | 6.761182458 |
| 19 | Fp2 | 459.153034 | 79.87834529 | 15.42956636 | 13.7559992 | 5.934348649 |
| 19 | AF8 | 718.0402135 | 78.75485374 | 16.09438516 | 27.33898184 | 21.91411139 |
| 19 | F3 | 2137.38603 | 140.5014383 | 39.94071996 | 57.07823286 | 22.12533021 |
| 19 | F4 | 1340.799519 | 146.9050087 | 57.72741376 | 129.3642469 | 73.91242422 |
| 19 | P3 | 521.2762999 | 71.29569842 | 15.62141435 | 17.11365124 | 7.649212252 |
| 19 | P4 | 520.8189024 | 72.18753271 | 19.43665534 | 38.84634867 | 23.94905178 |
| 19 | PO7 | 469.6533096 | 74.53028247 | 15.49515833 | 16.50661699 | 7.118294312 |
| 19 | O1 | 536.1748683 | 80.99231124 | 16.61391284 | 15.60462463 | 7.51618093 |
| 19 | O2 | 471.1216587 | 76.28735833 | 15.43535982 | 15.86185643 | 7.35487336 |
| 19 | PO8 | 444.0363291 | 70.916705 | 14.11415545 | 14.50425524 | 9.125903609 |
| 20 | AF7 | 233.3765254 | 49.55687813 | 9.790842613 | 10.39311874 | 3.938678253 |
| 20 | Fp1 | 229.4589627 | 51.19879192 | 10.83021412 | 7.232988567 | 2.502301912 |
| 20 | Fp2 | 222.1516034 | 52.16566299 | 10.63032896 | 5.766196316 | 1.700677802 |
| 20 | AF8 | 227.7317191 | 53.68277756 | 11.53970971 | 8.287332167 | 2.278315545 |
| 20 | F3 | 245.8652511 | 56.80187921 | 16.44077635 | 9.884161875 | 3.162883544 |
| 20 | F4 | 240.0579176 | 59.14052047 | 16.81208307 | 8.490260655 | 2.230537517 |
| 20 | P3 | 221.3290762 | 55.8734083 | 16.38101904 | 6.858344702 | 1.592603416 |
| 20 | P4 | 224.0222142 | 61.58544202 | 21.96859503 | 9.891435113 | 2.580155845 |
| 20 | PO7 | 324.1818978 | 95.07562136 | 25.10337468 | 17.61398827 | 4.269381484 |
| 20 | O1 | 212.4713054 | 61.61792839 | 14.61050617 | 10.25663447 | 2.547457565 |
| 20 | O2 | 224.7481571 | 60.76868552 | 17.1882184 | 11.12291778 | 3.039989056 |
| 20 | PO8 | 245.2101272 | 84.5750665 | 22.84834846 | 12.7600546 | 3.508389722 |
| 21 | AF7 | 505.8687822 | 57.52833173 | 12.65351903 | 15.90729028 | 5.505939402 |
| 21 | Fp1 | 490.7377943 | 58.65378087 | 7.912386387 | 12.36215691 | 5.257920573 |
| 21 | Fp2 | 201.8750467 | 29.72111717 | 8.995999882 | 13.3756287 | 5.251092598 |
| 21 | AF8 | 1042.412563 | 140.4107832 | 9.098137602 | 13.88346911 | 6.851473318 |
| 21 | F3 | 50.68768756 | 11.34566503 | 6.313875343 | 10.74782399 | 4.746621943 |
| 21 | F4 | 265.0226636 | 22.61732549 | 7.514992903 | 12.0406949 | 5.736400008 |
| 21 | P3 | 44.45208066 | 7.958671514 | 5.368387478 | 10.08610464 | 4.68749126 |
| 21 | P4 | 86.60689007 | 9.817574485 | 6.838224225 | 12.11378719 | 5.38228634 |
| 21 | PO7 | 252.3326118 | 19.14406619 | 6.937240901 | 12.35712233 | 5.861998787 |
| 21 | O1 | 68.41756185 | 20.19117413 | 13.70448497 | 20.23638573 | 7.320497075 |
| 21 | O2 | 43.58885154 | 10.26910974 | 8.58484866 | 15.91535266 | 7.254322995 |
| 21 | PO8 | 125.0955593 | 21.12539814 | 10.17253932 | 18.0949808 | 7.975178163 |
| 22 | AF7 | 105.0617352 | 15.5858292 | 4.387534164 | 9.064939789 | 3.723701043 |
| 22 | Fp1 | 77.70028544 | 14.36295307 | 4.780629323 | 8.9054272 | 2.563185823 |
| 22 | Fp2 | 72.16859304 | 13.8908467 | 3.895631948 | 7.453956377 | 1.956523614 |
| 22 | AF8 | 82.397128 | 14.82627172 | 4.390709683 | 9.773824541 | 2.999797853 |
| 22 | F3 | 194.5168711 | 22.99053627 | 6.861474774 | 12.47428899 | 2.458626729 |
| 22 | F4 | 113.1345407 | 16.95351673 | 5.369847506 | 11.86893904 | 2.423900822 |
| 22 | P3 | 209.0776414 | 22.01437566 | 5.532132063 | 7.823689369 | 2.041767136 |
| 22 | P4 | 73.00063757 | 14.11832523 | 4.726944865 | 8.587481955 | 2.050427134 |
| 22 | PO7 | 692.0260166 | 56.93361659 | 9.268221045 | 10.11046969 | 3.226650086 |
| 22 | O1 | 1088.342992 | 48.000841 | 16.74286713 | 15.94216059 | 3.913367124 |
| 22 | O2 | 73.13902589 | 15.76866459 | 6.810685771 | 8.279200563 | 2.293410647 |
| 22 | PO8 | 87.62519776 | 27.54516789 | 9.127702105 | 10.33972507 | 3.376273464 |

**FIGURE S1.** Python script used for EEG data analysis and reproducibility.


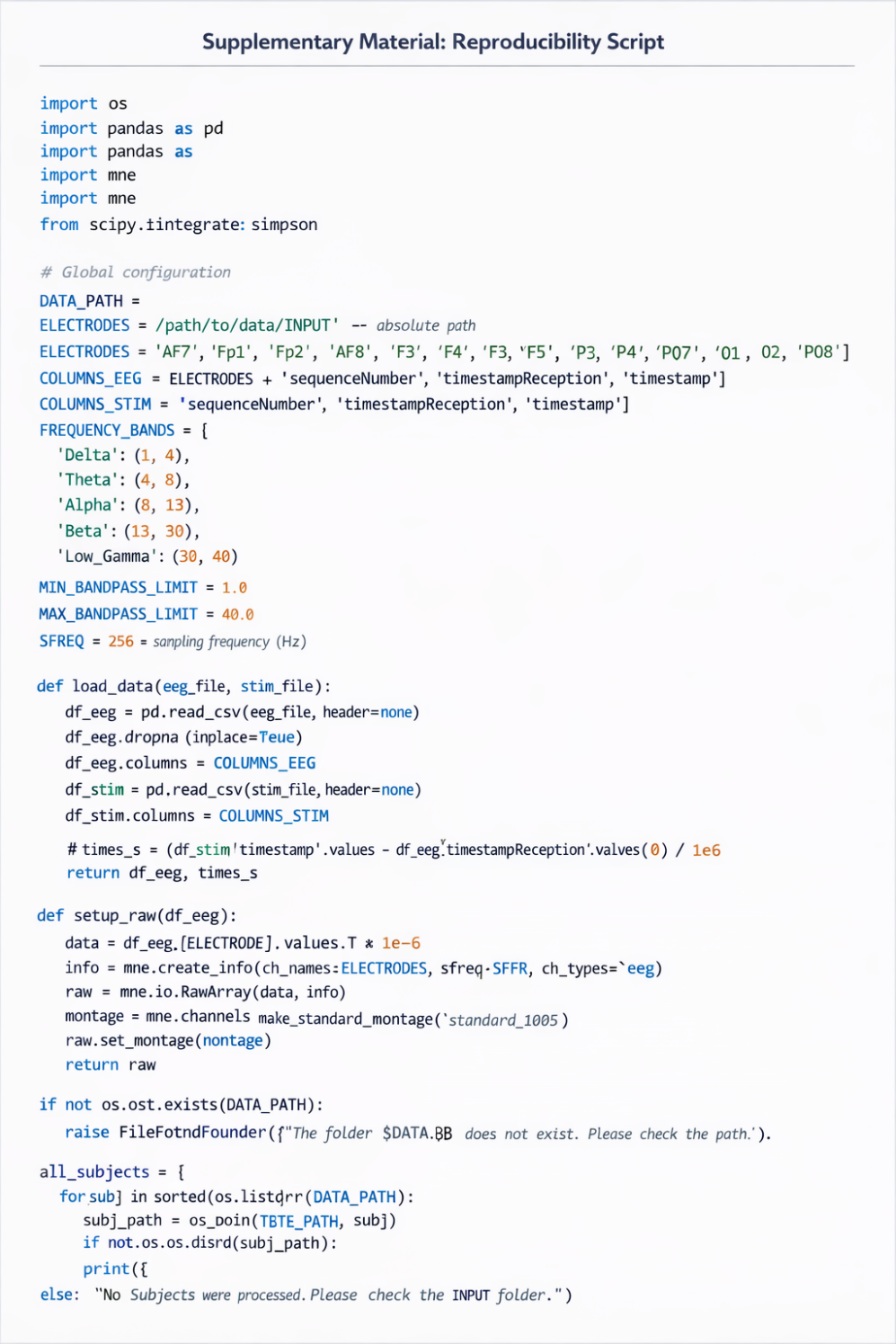


Note. The script illustrates the full processing pipeline, including data loading, signal preprocessing, power spectral density estimation using Welch’s method, and computation of absolute band power in canonical frequency bands (Delta, Theta, Alpha, Beta, and low Gamma low) for each EEG channel and participant.
